# Dental crown morphological variation and heterodonty in carcharhiniform sharks

**DOI:** 10.1002/ar.70111

**Published:** 2025-12-15

**Authors:** Flávia Zanini, Karla D. A. Soares

**Affiliations:** ^1^ Departamento de Zoologia, Instituto de Biociências Universidade de São Paulo São Paulo Brazil; ^2^ Departamento de Zoologia, Instituto de Biologia Universidade Federal do Rio de Janeiro Rio de Janeiro Brazil

**Keywords:** comparative anatomy, dentition, morphology, sharks

## Abstract

Elasmobranch teeth are highly mineralized structures that constitute the majority of the fossil record for this group. Despite their taxonomic and evolutionary significance, detailed descriptions of dental morphology remain scarce. The order Carcharhiniformes, the most diverse among sharks, comprises 304 valid species that display remarkable diversity and complexity in dentition. This study aims to describe and illustrate crown morphological variation within Carcharhiniformes, with a focus on family‐level patterns and types of heterodonty. Tooth samples were obtained from specimens preserved in 70% ethanol and from dry jaws. A total of 66 samples, representing all families except Dichichthyidae, were examined and photographed using a scanning electron microscope (SEM). The types of heterodonty considered include dignathic, gynandric, monognathic, and ontogenetic. Dignathic and monognathic heterodonty were further classified as weak, moderate, and strong. Four sub‐arrangements were recognized: imbricate, independent, mixed, and overlap, as well as the pavement and independent dentition arrangements. New data reveal previously unrecognized patterns of variation across the four types of heterodonty in Carcharhinidae, Scyliorhinidae, Proscylliidae, Triakidae, and Pentanchidae. High‐resolution SEM images allowed detailed characterization of crown morphology, highlighting subtle diagnostic features and family‐level differences across heterodonty types. Dental complexity in Carcharhiniformes is largely driven by the distribution and combination of heterodonty types. These detailed dental descriptions provide a valuable framework for understanding the diversity and evolutionary significance of dentition within the most diverse order of sharks.

## 1 INTRODUCTION

Chondrichthyes possess highly mineralized teeth, in contrast to their cartilaginous and low calcified skeletons. Elasmobranchii (sharks, skates, and rays) exhibit polyphyodont dentitions with lyodont teeth, producing thousands of teeth throughout their lives. Due to their strong mineralization, abundance, and preservation potential, teeth are the most commonly encountered remains in the shark fossil record (Cappetta, 2012; Compagno, 1988; Enax et al., 2012; Grady, 1970; Guinot et al., 2018; Maisey, 1984; Schnetz et al., 2016; Springer & Gold, 1989; White, 1937). Consequently, the morphology of fossil shark teeth has been studied in greater detail than that of teeth from extant species. However, as emphasized by Agassiz (1835–1843), greater attention to the dentition of living species is essential. Dental features not only contribute to the accurate description of species but also hold the potential to shed light on the taxonomy and diversity of extinct taxa and the evolutionary history of shark orders (Cappetta, 1986, 2012; Compagno, 1988; Guinot et al., 2018; Mollen, 2019; Schnetz et al., 2016; White, 1937).

Carcharhiniformes represents the most diverse order of sharks, comprising 12 families, 52 genera, and 304 valid species (Ebert et al., 2013; Fricke et al., 2025) that occur in all oceans except the Southern Ocean. These sharks inhabit a wide range of environments, from shallow coastal waters to deep oceanic regions, reaching depths of about 2000 meters (Compagno, 1988; Ebert et al., 2021). Within this order, considerable variation exists in body size and ecological strategies, ranging from small, demersal species such as catsharks to large, pelagic species like those in the families Carcharhinidae and Sphyrnidae. This ecological and behavioral diversity is mirrored by significant variation in dentition, characterized by distinct tooth arrangements, ornamentations, and heterodonty (dignathic, monognathic, gynandric, and ontogenetic; Compagno, 1988; López et al., 2006; Nelson, 2006; Nelson et al., 2016; Ebert et al., 2021). Furthermore, in addition to interspecific variation, there is considerable intraspecific and populational‐level variation, as well as dental deformities. Consequently, comparative studies of carcharhiniform dentitions are challenging (Compagno, 1979, 1988; Guinot et al., 2018).

Despite the taxonomic and evolutionary importance of dental morphology, detailed descriptions of shark dentitions remain scarce. The lack of such data can lead to misidentifications and taxonomic inaccuracies, as highlighted by numerous reclassifications of fossil and extant species. For example, several fossil species initially assigned to the genus *Scyliorhinus* likely belong to other taxa. This reclassification resulted from the identification of morphological differences in dental structures between extant and fossil species, thereby reducing the taxonomic heterogeneity of *Scyliorhinus* (Cappetta, 2012). Adult males belonging to the genera *Rhizoprionodon* and *Scoliodon* were previously classified within the genus *Physodon*. However, the dental characteristics used to define *Physodon* were later understood to represent gynandric heterodonty (Maisey, 1984). Similarly, species of *Carcharhinus*, such as *C. limbatus* (Valenciennes, 1839) and *C. brevipinna* (Valenciennes, 1839), also have a problematic taxonomic history due to poorly understood characteristics of ontogenetic heterodonty. Consequently, juvenile and adult specimens were assigned to distinct genera (Garrick, 1982). The genus *Triakis*, proposed by Müller & Henle (1838), was initially distinguished from *Mustelus* solely by dental morphology. Later, many species were included in *Triakis* due to dental similarities, turning the genus into a wastebasket taxon for a period (Compagno, 1988). Another example involving living species is the co‐occurrence of the catshark *Apristurus manis* (Springer, 1979) and *A. microps* (Gilchrist, 1922) in the Atlantic Ocean. The original descriptions of both species, as well as subsequent publications, do not provide features that allow a putative distinction in the field, nor do they properly illustrate or describe their dental characteristics. However, unpublished data suggest that dental morphology could serve as a useful diagnostic feature (Guinot et al., 2018).

Advancements in imaging technologies, such as scanning electron microscopy (SEM), have enabled more detailed analyses of tooth morphology. Yet, studies employing these methods remain limited, and attention to the details of dental morphology has progressively declined (Cappetta, 2012; Guinot et al., 2018; Maisey, 1984). Guinot et al. (2018) reported that only 37% of shark species have adequately documented dental features. This gap underscores the need for comprehensive studies that integrate high‐quality imaging with detailed morphological descriptions (Cappetta, 2012; Gomes, 1988; Guinot et al., 2018; Maisey, 1984).

Therefore, this study aims to describe and illustrate morphological variation in dental crowns of Carcharhiniformes, focusing on family‐level patterns and types of heterodonty. By addressing these gaps, we hope to improve the understanding of dental diversity within this order and its implications for taxonomy, systematics, and paleontology.

## 2 MATERIALS AND METHODS

Tooth samples were obtained from specimens preserved in 70% ethanol and from dry jaws deposited in the following institutions: BMNH, Natural History Museum, London; MNRJ, Museu Nacional, Rio de Janeiro; MZUSP, Museu de Zoologia da Universidade de São Paulo, São Paulo; NSMT, National Museum of Nature and Science, Tsukuba; SAIAB, South African Institute for Aquatic Biodiversity, Grahamstown; UERJ, Universidade do Rio de Janeiro, Rio de Janeiro; UF, Florida Museum of Natural History, Gainesville; USNM, National Museum of Natural History, Smithsonian Institution, Washington D.C. Abbreviations follow Sabaj (2020). A total of 66 samples, representing 27 genera and 45 species from all families, except Dichichthyidae (White et al., 2024), of the order Carcharhiniformes, was examined. A complete list of material examined is presented below; family followed by genera, species name, the institution acronym, catalog number, sex, total length, in millimeters, and locality, when information is available.

Atelomycteridae: *Atelomycterus fasciatus*, MZUSP 118095, female, 363 mm TL (Western Australia); *Schroederichthys bivius*, BMNH 1999.5.4.5, male, 680 mm TL (Falkland Islands), USNM 114731, male, 600 mm TL (41°54′S, 73°06′O); *S. chilensis*, USNM 221652, female, 525 mm TL (39°42′S, 73°27′O); *S. maculatus*, USNM 187690, male, 325 mm TL (14°10′N, 81°58′O); *S. saurisqualus*, UERJ 944, female, 606.2 mm TL (São Paulo, Brazil), UERJ not cataloged, male, 597 mm TL (São Paulo, Brazil). Carcharhinidae: *Carcharhinus brevipinna*, AC.UERJ 298, no sex and TL data (Niterói, Rio de Janeiro, Brazil), AC.UERJ 301, no sex and TL data (Atafona, Rio de Janeiro, Brazil); *C*. cf. *falciformis*, AC.UERJ 1453, female, no TL data, AC.UERJ 1458, male, no TL data (Arquipélago de São Pedro São Paulo, Rio Grande do Norte, Brazil); *C. limbatus*, AC.UERJ 295, male, 1500 mm TL (Itaipu, Rio de Janeiro, Brasil); *C. plumbeus*, AC.UERJ 092, no sex and TL data (Atafona, Rio de Janeiro, Brazil); *C. signatus*, UERJ 2023, female, no TL and locality data, AC.UERJ 889, no sex and TL data (Santos, São Paulo, Brazil), AC.UERJ 890, no sex and TL data (Santos, São Paulo, Brazil); *Negaprion brevirostris*, MNRJ 16588, female, no TL data (Natal, Rio Grande do Norte, Brazil); *Rhizoprionodon lalandii*, MZUSP 037300, female, 330 mm TL (Baía da Ilha Grande, Rio de Janeiro, Brazil), MZUSP 037300, male, 320 mm TL (Baía da Ilha Grande, Rio de Janeiro, Brazil), MZUSP not cataloged, female, 605 mm TL (Cananéia, São Paulo, Brazil), MZUSP not cataloged, male, 525 mm TL (Cananéia, São Paulo, Brazil), USP not cataloged, female, 355 mm TL (no locality data); *R. porosus*, MNRJ 33828, male, 600 mm TL (no locality data), MZUSP 060555, female, 365 mm TL (Arquipélago dos Abrolhos, Bahia, Brazil), MZUSP 060555, male, 370 mm TL (Arquipélago dos Abrolhos, Bahia, Brazil), MZUSP not cataloged, female, 450 mm TL (Cananéia, São Paulo, Brazil), MZUSP not cataloged, male, 470 mm TL (Cananéia, São Paulo). Galeocerdonidae: *Galeocerdo cuvier*, MZUSP 112027, female, no TL and locality data, MNRJ not cataloged, no sex and TL data (São João da Barra, Rio de Janeiro). Hemigaleidae: *Paragaleus pectoralis*, USNM 197626, male, 470 mm TL (Lagos, Nigeria). Leptochariidae: *Leptocharias smithii*, MNRJ 46762, male, 620 mm TL (Kongo‐Central). Pentanchidae: *Apristurus australis*, MZUSP 118094, female, 493 mm TL (33°49′S, 151°57′L); *A. laurussonii*, UF 185652, male, 480 mm TL (Gulf of Mexico); *A. parvipinnis*, USNM 221497, male, 230 mm TL (29°0.5′N, 88°22′O, 732 meters deep); *Bythaelurus lutarius*, SAIAB 81853, male, 290 mm TL (25°34.5′S, 37°13′L); *Cephalurus* sp., USNM 221527, female, 285 mm TL (Peru); *Figaro boarmani*, MZUSP 118096, male, 486 mm TL (35°55.6′S, 150°1.9′L); *Galeus antillensis*, UF 14824, no sex and TL data (Honduras); *G. melastomus*, USP not cataloged, female, 370 mm TL (no locality data); *G. mincaronei*, USP not cataloged, female, 295 mm TL (no locality data); *G. polli*, USNM 221610, male, 378 mm TL (35°41′N, 4°55′O); *Halaelurus natalensis*, SAIAB 26951, male, 400 mm TL (South Africa); *Haploblepharus edwardsii*, USNM 226008, no sex and TL data (Cape Town, South Africa); *Holohalaelurus regani*, SAIAB 25717, male, 610 mm TL (South Africa); *Holohalaelurus* sp., MZUSP not cataloged, male, no TL data (South Africa). Proscylliidae: *Eridacnis barbouri*, USNM 188023, male, 265 mm TL (23°59′N, 79°17′O). Pseudotriakidae: *Gollum attenuatus*, NSMT 42855, female, 950 mm TL (New Zealand). Scyliorhinidae: *Cephaloscyllium sufflans*, SAIAB 6242, male, 800 mm TL (South Africa); *C. umbratile*, USP not cataloged, male, 409 mm TL (Taiwan); *Poroderma africanum*, SAIAB 25343, male, 920 mm TL (Cape Town, South Africa); *P. pantherinum*, SAIAB 34577, male, 640 mm TL (Cape Town, South Africa); SAIAB 25921, male, no TL data (South Africa); *Scyliorhinus cabofriensis*, USNM 221611, male, 522 mm TL (Barbados), USP not cataloged, male, 425 mm TL; *S. capensis*, SAIAB 40762, male, 325 mm TL (34°10′S, 23°22′L), SAIAB 12160, female, 320 mm TL (Algoa Bay, South Africa); *S. ugoi*, USP not cataloged, male, 525 mm TL (Brazil). Sphyrnidae: *Sphyrna lewini*, AC UERJ 102, male, 1192 mm TL (Rio de Janeiro, Brazil), MZUSP 2463, female, 690 mm TL (Cananéia, São Paulo, Brazil), MNRJ 39095, male, 645 mm TL (no locality data). Triakidae: *Galeorhinus galeus*, UERJ 291, male, 498 mm TL (Tramandaí, Rio Grande do Sul, Brazil); *Hemitriakis japanica*, NSMT 66856, female, 515 mm TL (Japan); *Iago omanensis*, USNM 326606, male, 350 mm TL (Israel); *Mustelus canis*, AC.UERJ 088, no sex, TL and locality data, USP not cataloged, two females, 685–795 mm TL (no locality data); *Mustelus schimitti*, MZUSP not cataloged, female 620 mm TL, male, 670 mm TL (Cananéia, São Paulo, Brazil); *Triakis semifasciata*, UERJ 1610.1, male, 413 mm TL (California, United States).

Tooth samples were taken from anterior to commissural portions of upper and lower jaws, preferably from the right side, boiled for 5 min, and submerged in a pancreatin solution for six to 24 h to dissociate teeth from connective tissue. Tooth groups were identified according to their relative positions along the jaws and separated from the gum with the aid of a stereomicroscope. Nomenclature for tooth groups follows Herman et al. (1987, 1991), Cappetta (2012).

Teeth were photographed using the scanning electron microscope ZEISS SIGMA VP and ZEISS LEO 440, housed, respectively, in the Departamento de Zoologia and Museu de Zoologia of the Universidade de São Paulo. SEM photographs were edited with the aid of Adobe Photoshop CS6.

The descriptions presented herein are primarily grounded in direct observations of the examined taxa. Nevertheless, data from the literature, particularly concerning the analyzed species, was also reviewed and integrated when relevant.

### 2.1 Nomenclature

Nomenclature for dentition follows Compagno (1970, 1988, 2001), Gomes (1988), Herman et al. (1987, 1991), Cappetta (2012), and Soares & de Carvalho (2019). Terminology and definitions of tooth arrangements were compiled from Strasburg (1963), Springer (1966) and Compagno (1988). Row and series definitions follow Compagno (1988). Tooth root was not considered in this study.

Compagno (1970, 1988, 2001) described the basal ledge as a shelf‐like projection on the outer surface of the crown base that overlaps the basal groove. Compagno (1970, 1988) considered the basal groove as part of the root and the basal ledge as part of the crown. However, both the basal groove and the basal ledge are in the enamel‐covered region. Furthermore, the basal groove and basal ledge are related structures. For this reason, the basal groove and the ledge are considered as one structure in the present study. The term ‘ectodermal pits’ was first described by Hertwig (1874) to refer to concavities observed in dermal denticles (Raschi & Tabit, 1992). Soares & de Carvalho (2019) observed similar structures in the teeth of *Scyliorhinus*. Since denticles and teeth are homologous structures, and the terms used by authors like Herman et al. (1991) to refer to these structures in teeth are vague, Soares & de Carvalho (2019) used the term ‘ectodermal pits’ also to refer to the impressions found in the teeth. A list of dental regions and structures, their definitions and synonyms is presented in Table 1.

**TABLE 1:** Anatomical terms used in this study with their definitions and synonyms.

Terminology	Description	Synonyms
Basal groove	Groove circling the tooth between crown base and root	Basal ledge (Compagno, 1970, 1988, 2001)
Crown base	Most basal and laterally expanded region of the crown	Base (Applegate, 1965; Arambourg, 1952; Bigelow & Schroeder, 1948; Cappetta, 1981, 1990; Chapman, 1918; Dean, 1909; Eastman, 1908; Fournier & Pruvost, 1928; Garman, 1913; Kemp, 1978; Leriche, 1905; Longbottom, 1979; Patterson, 1966; Rebouças & Silva Santos, 1956; Reif, 1977; Richter, 1987; Silva Santos & Salgado, 1971; Springer, 1966; White, 1937; Williston, 1900) Crown foot (Compagno, 1970, 1988, 2001; Gomes, 1988) Enamel base (Ameghino, 1908) Flange (Seigel, 1978) Trunk (Thies & Reif, 1985)
Distal region	Tooth region toward commissure of the jaw	Commissural (Antunes & Jonet, 1970; Arambourg, 1952; Gomes, 1988; Raschi et al., 1982) External (Cappetta, 1981; Pequeño, 1977) Lingual (Hotton, 1952) Outer (Bigelow & Schroeder, 1948; Garman, 1913; Springer, 1938) Posterior (Ameghino, 1908; Antunes & Jonet, 1970; Leriche, 1905; Silva Santos & Salgado, 1971) Postlateral (Compagno, 1970)
Ectodermal pits	Microscopic oval impressions on the crown surface; collectively, impressions form a reticulated pattern	Reticulated ornamentation (Herman et al., 1991)
Lateral expansion	Rounded edge expansions projected laterally to principal cusp. Could be cutting or blunt	Basal expansions, basal sector (Bigelow & Schroeder, 1948), blade (Cappetta, 2012; Herman et al., 1991), commissural process (Seigel, 1978), commissural/symphyseal projection (Gomes, 1988), lateral extension (Longbottom, 1979), jagged blade (Compagno, 1988), heel (Cappetta, 2012), shoulder (Garman, 1913; Compagno, 1970, 1988, 2001), talon (Leriche, 1905; Arambourg,^1952^; Antunes & Jonet, 1970; Cappetta, 1970, 1981, 1990)
Limiting zone	Finely porous region between the most basal portion of enameloid (crown) and the uppermost portion of the osteodentine (root) that surrounds the tooth without strangling it	Pad of enamel (Longbottom, 1979) Neck (Compagno, 1988, 2001)
Mesial region	Tooth region toward symphysis of the jaw	Anterior (Ameghino, 1908; Antunes & Jonet, 1970; Leriche, 1905; Rebouças & Silva Santos, 1956; Silva Santos & Salgado, 1971); inner (Bigelow & Schroeder, 1948; Garman, 1913; Nakaya, 1975), internal (Cappetta, 1981), labial (Hotton, 1952), premedial (Compagno, 1970), sinfisial/symphyseal (Arambourg, 1952; Gomes, 1988; Raschi et al., 1982)
Principal cusp	Higher cusp of the crown, situated on the central region of the crown, or slightly laterally dislocated. Longitudinal axis oblique or perpendicular to the crown base	Acrocone (Cappetta, 1981, 1990), anterior cusp (Chapman, 1918), axial cusp (Bigelow & Schroeder, 1948), central cusp (Bigelow & Schroeder, 1948; Dean, 1909; Kemp, 1978; Patterson, 1966; Richter, 1987; Springer, 1966; White, 1937; Williston, 1900), central cone (Chapman, 1918; Eastman, 1908), cusp (Cappetta, 1981; Cappetta, 2012; Compagno, 2001; Garman, 1913; Hotton, 1952; Raschi et al., 1982; Seigel, 1978; Springer, 1938; Williston, 1900), dentículo principal (Gomes, 1988), main cusp (Heemstra, 1973; Reif, 1977; Springer, 1938; Thies & Reif, 1985; Williston, 1900), median cusp (Bigelow & Schroeder, 1948; Cappetta, 1990; Compagno, 1977; Garman, 1913; White, 1937; Williston, 1900), median cone (Applegate, 1967; Chapman, 1918; Eastman, 1908), middle cusp (Williston, 1900), middle/median denticle (Eastman, 1908; Fournier & Pruvost, 1928), primary cusp (Arambourg, 1952; Bass et al., 1973; Bigelow & Schroeder, 1948; Compagno, 1970; Compagno, 1988; Garman, 1913; Kemp, 1978; Nakaya, 1975), principal cone (Chapman, 1918; Eastman, 1908; Fournier & Pruvost, 1928; Silva Santos & Salgado, 1971; Williston, 1900)
Protuberances	Rounded projections present on the crown base margin	
Serration	Triangular‐like modification in the cutting edge, the apex of these modifications can be more rounded or pointed; denticulate cutting edges	Basal denticles (Kemp, 1978), crenulation (Ameghino, 1908; Antunes & Jonet, 1970; Arambourg, 1952; Longbottom, 1979), cusplets (Raschi et al., 1982), denticulation (Silva Santos & Salgado, 1971), rounded denticles (Richter, 1987)
Striae	Vertically elongated prominences, generally beginning at the crown base and extending toward the crown apex. The extension of the striae is variable	Grooves (Chapman, 1918), pleats (Rebouças & Silva Santos, 1956; Antunes & Jonet, 1970; Cappetta, 1990), ribs (Reif, 1977), ridges (Compagno, 1977; Springer, 1966; Williston, 1900), sculpture (Compagno, 1977), transverse ridges (Cappetta, 1990; Compagno, 1970, 1988, 2001), vertical ridges (Cappetta, 2012), vertical striae (Leriche, 1905), vertical striations (Longbottom, 1979), vertical wrinkles (Arambourg, 1952; Williston, 1900)

### 2.2 Types of heterodonty

Heterodonty types are defined as follows: *dignathic heterodonty* (Figure 1)—tooth differences in shape or size observed when upper and lower jaws are compared; present along with the dental series or only in some tooth groups; *gynandric heterodonty* (Figure 2) (or sexual heterodonty; Herman et al., 1991; Cappetta, 2012; Soares & de Carvalho, 2019)—tooth differences between males and females of the same species and at about the same developmental stage; *monognathic heterodonty* (Figure 3), tooth differences in shape or size along a jaw series (upper or lower); *ontogenetic heterodonty* (Figure 4), gradual changes in dental morphology during individual growth. The ‘size heterodonty’ of Gomes (1988), characterized by teeth in the same region of the upper and lower jaws differing only in size, is incorporated into the definitions of dignathic and monognathic heterodonty presented here.

**FIGURE 1:**
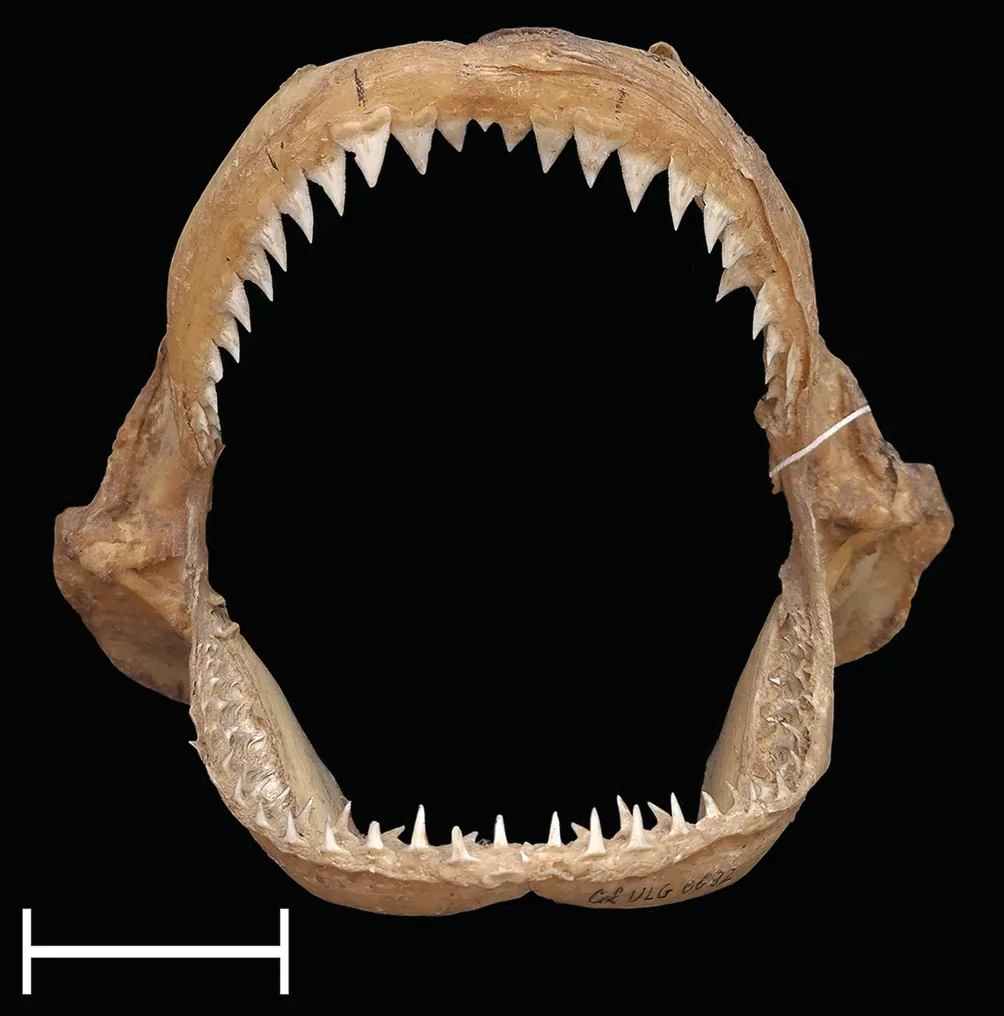
Dignathic heterodonty. Upper and lower jaws of *Carcharhinus plumbeus*. AC.UERJ 092, no sex and TL data. Scale bar = 5 cm.

**FIGURE 2:**
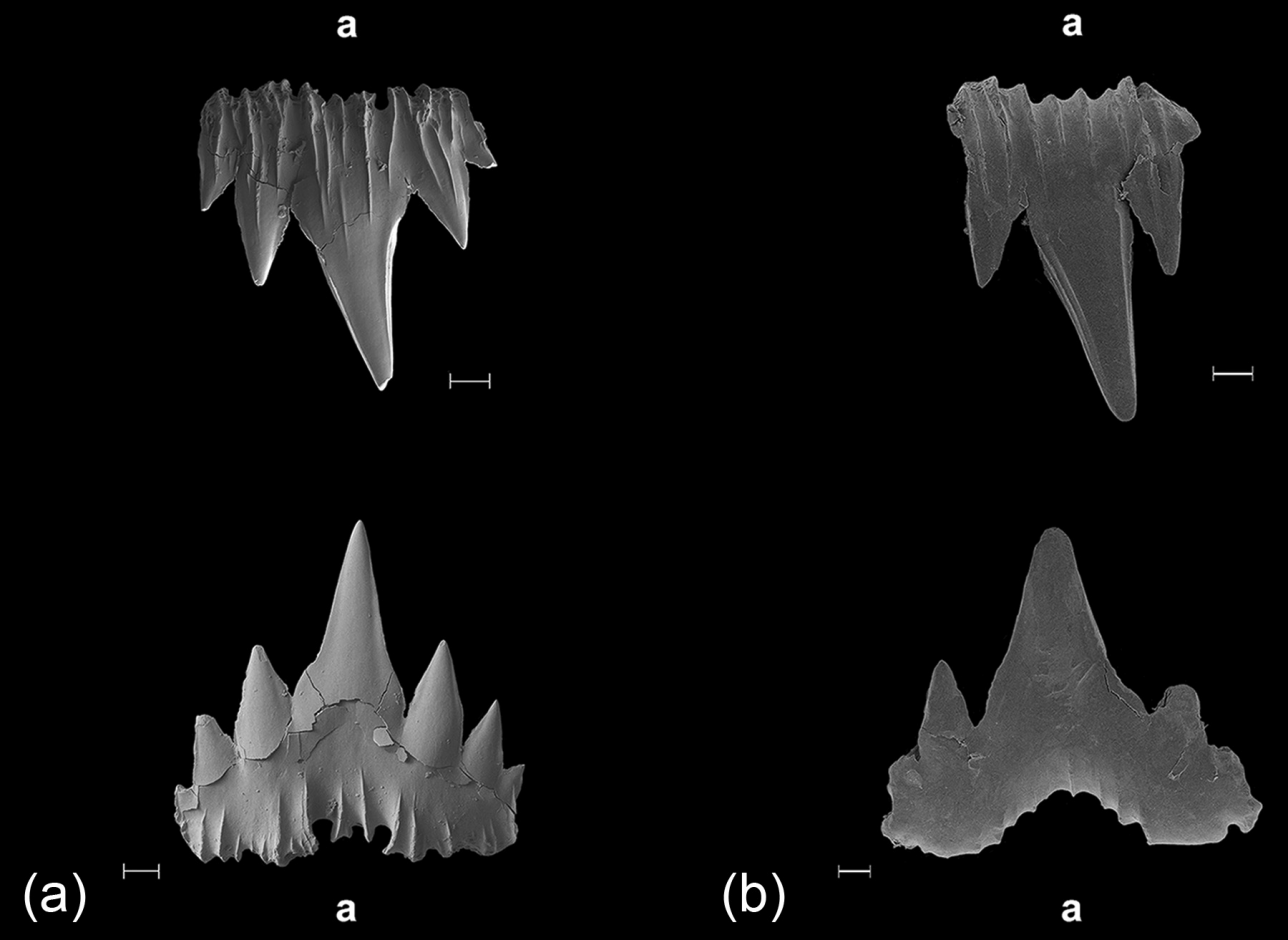
Gynandric heterodonty. Labial face of anterior teeth of *Schroederichthys saurisqualus*. (a) UERJ 944, female, 606.2 mm TL. (b) UERJ not cataloged, male, 597 mm TL. Scale bars = 100 μm. a, anterior. Modified from the work of Soares & Zanini (2023).

**FIGURE 3:**
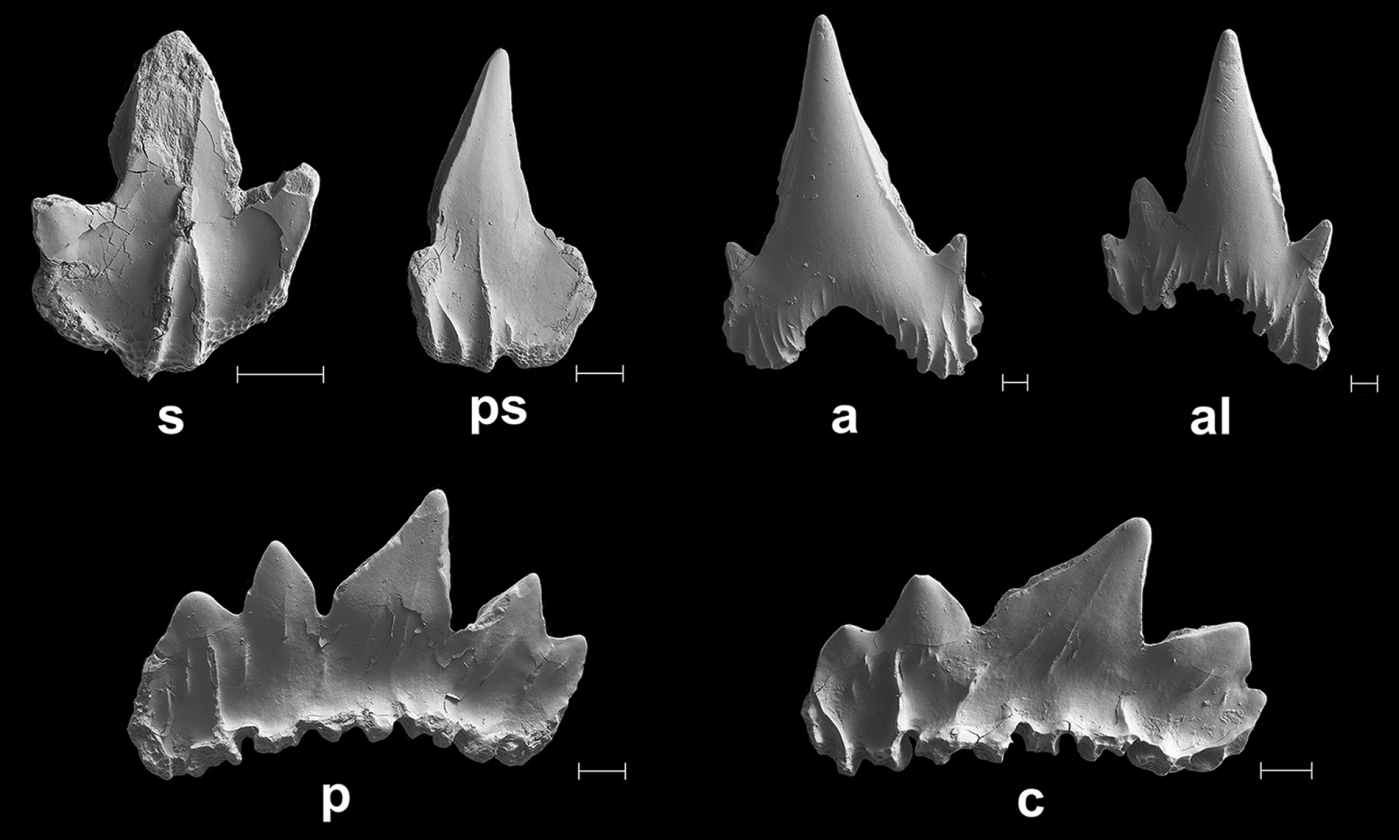
Monognathic heterodonty. Labial face of teeth of a male specimen of *Holohalaelurus regani*. SAIAB 25717, 610 mm TL. Scale bars = 100 μm. a, anterior; al, anterolateral; c, commissural; p, posterior; ps, parasymphyseal; s, symphyseal.

**FIGURE 4:**
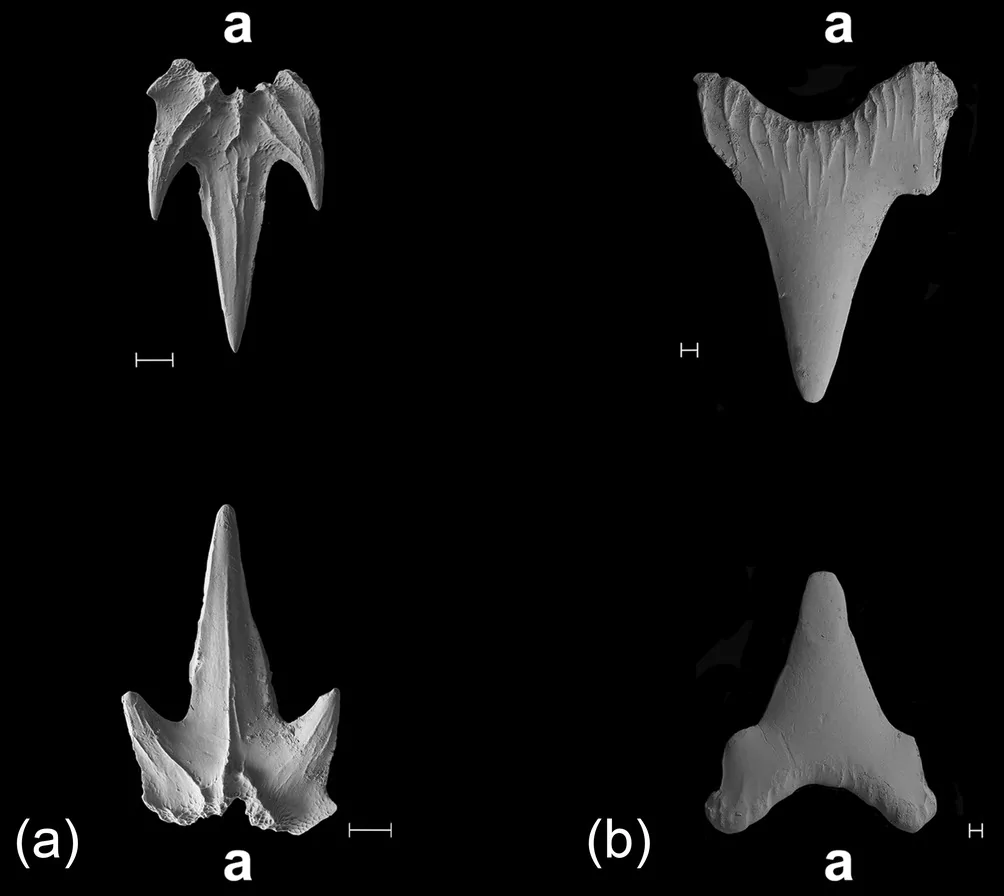
Ontogenetic heterodonty in *Scyliorhinus capensis*; labial face of anterior teeth. (a) SAIAB 12160, juvenile female, 320 mm TL. (b) SAIAB 26440, adult female, 843 mm TL. Scale bars = 100 μm. a, anterior. b, modified from the work of Soares & de Carvalho (2019).

Dignathic and monognathic heterodonty are classified into three levels: weak, moderate, and strong. In weak heterodonty, teeth are similar, with differentiation primarily in size, either along the same jaw or between the upper and lower jaws (e.g., *Sphyrna lewini*, Figure 5). In moderate heterodonty, teeth show noticeable, but not abrupt, differentiation along the same jaw or between the upper and lower jaws (e.g., upper teeth of *Leptocharias smithii*, Figure 6). Strong heterodonty refers to cases where teeth are significantly or entirely different between the anterior and commissural regions of the same jaw or between the upper and lower jaws (e.g., *Paragaleus pectoralis*, Figure 7).

**FIGURE 5:**
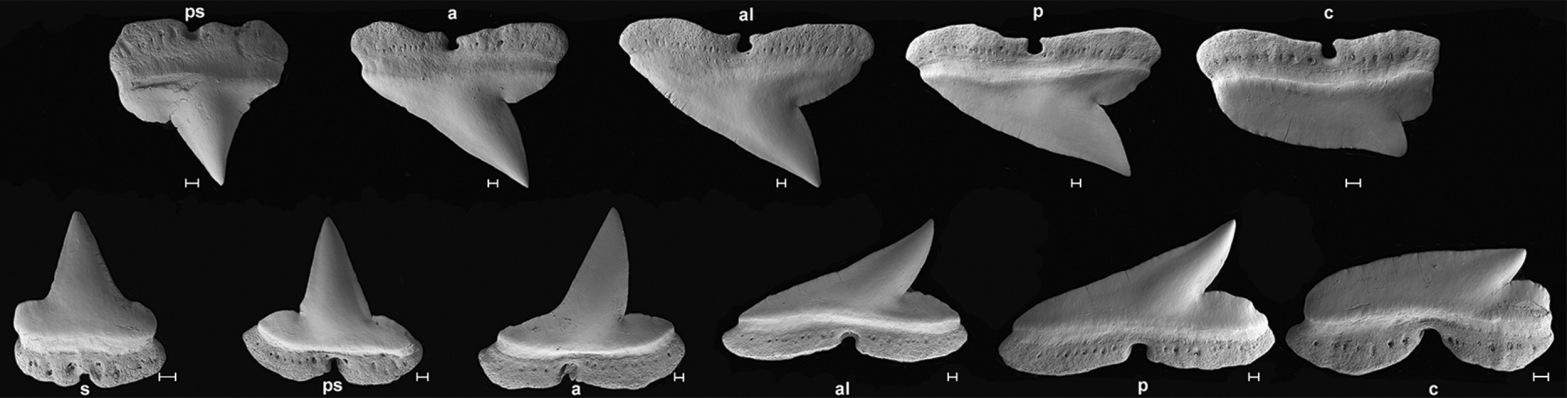
Labial face of teeth of a female specimen of *Sphyrna lewini*. MZUSP 2463, 690 mm TL. Scale bars = μ100 m. a, anterior; al, anterolateral; c, commissural; p, posterior; ps, parasymphyseal; s, symphyseal.

**FIGURE 6:**
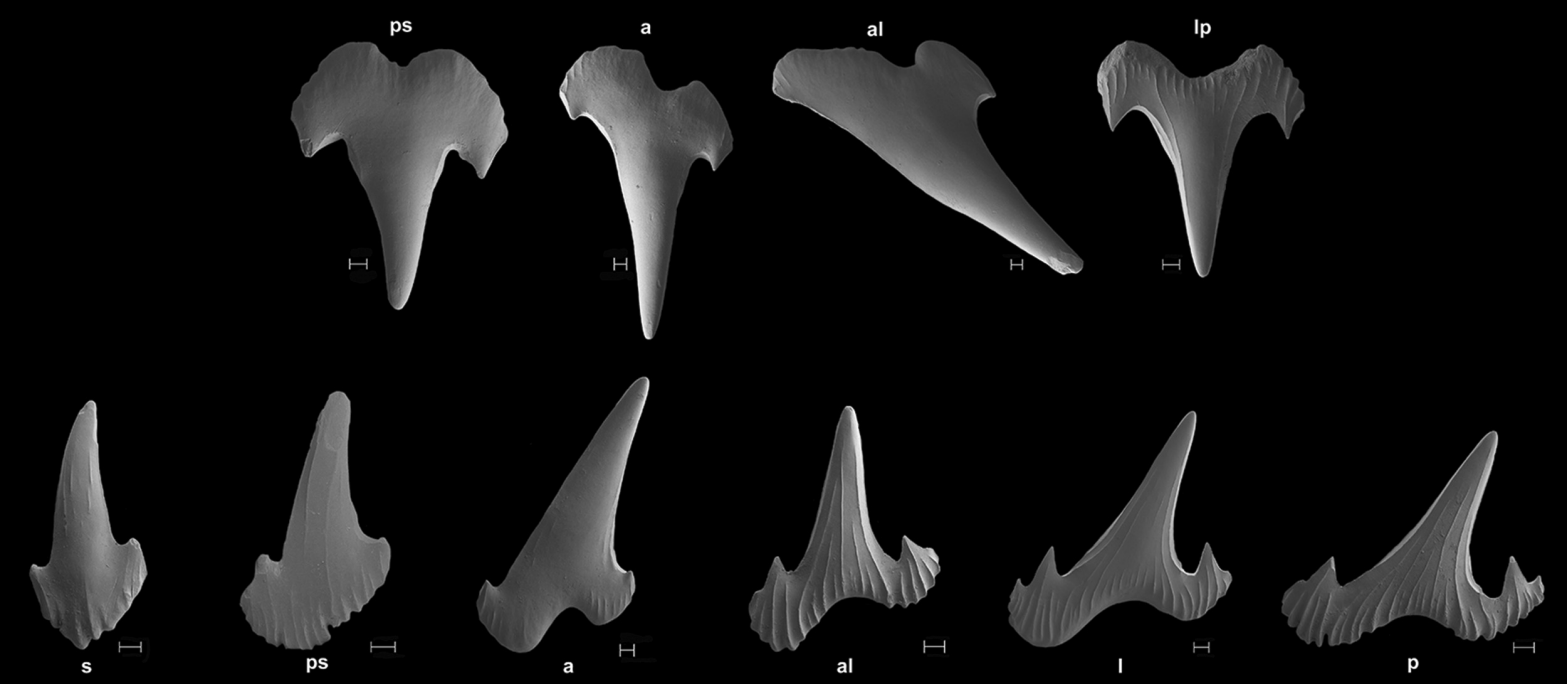
Labial face of teeth of a male specimen of *Leptocharias smithii*. MNRJ 46762, 620 mm TL. Scale bars = 100 μm. a, anterior; al, anterolateral; l, lateral; lp, lateroposterior; p, posterior; ps, parasymphyseal; s, symphyseal.

**FIGURE 7:**
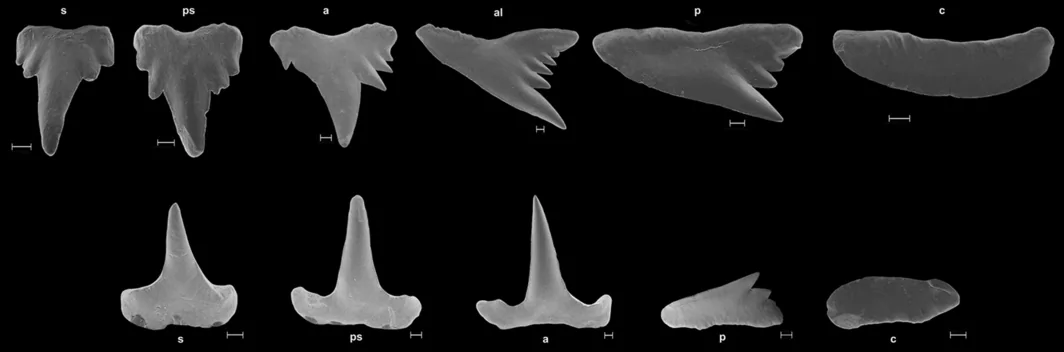
Labial face of teeth of a male specimen of *Paragaleus pectoralis*. USNM 197626, 470 mm TL. Scale bars = 100μ m. a, anterior; al, anterolateral; c, commissural; p, posterior; ps, parasymphyseal; s, symphyseal.

### 2.3 Arrangements and sub‐arrangements

Strasburg (1963) proposed five arrangements based on tooth replacement in sharks and the relationship among adjacent teeth in a series: alternate overlap, imbricate overlap, independent dentition, mixed alternate and imbricate overlap, and modified imbricate overlap—proposed exclusively for the genus *Isistius* (Lamniformes). However, an alternating arrangement in Carcharhiniformes primarily reflects the pattern of dental development and replacement present in the order (Smith et al., 2012; Smith et al., 2018; Underwood et al., 2015). Therefore, the Strasburg (1963) arrangements are proposed here as sub‐arrangements and described below.

1. *Alternate dentition arrangement*: in a single dental series, teeth are arranged alternately, with one tooth positioned slightly more labially, the adjacent tooth more lingually, and so on.
2. *Imbricate sub‐arrangement* (Figure 8a): mesial or distal edge of a tooth overlapped the mesial or distal edge of the adjacent tooth within the series.
3. *Independent sub‐arrangement* (Figure 8b): no overlap between teeth.
4. *Mixed sub‐arrangement* (Figure 8c): overlap and imbricate sub‐arrangements are observed along a series.
5. *Overlap sub‐arrangement* (Figure 8d): mesial and distal edges of more internal teeth in the series are overlapped by the mesial or distal edges of more external ones.
6. *Pavement dentition arrangement* (Figure 8e): adjacent teeth are very close to each other, frequently in touch but without overlapping; tightly arranged in a quincunx pattern. This arrangement does not typically form distinct series, and it is usually observed in representatives of *Mustelus*.

**FIGURE 8:**
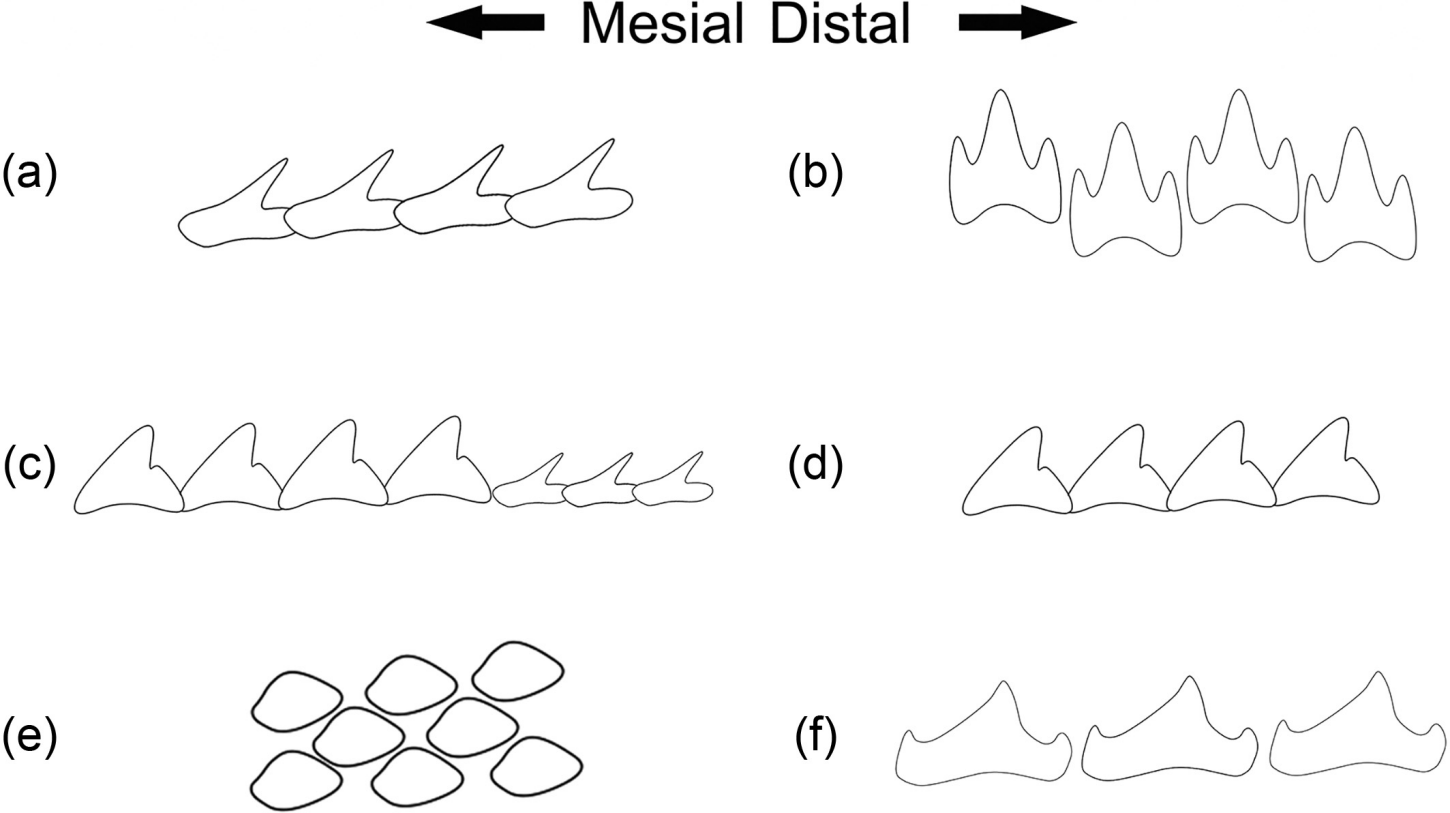
Tooth arrangements and sub‐arrangements. (a) Imbricate sub‐arrangement. (b) Independent sub‐arrangement. (c) mixed sub‐arrangement. (d) overlap sub‐arrangement. (e), Pavement dentition arrangement. (f) parallel dentition arrangement.

## 3 RESULTS

A new dentition arrangement was observed in the posterior and commissural tooth groups of *Schroederichthys chilensis* (Guichenot, 1848), family Atelomycteridae, and *Rhizoprionodon lalandii* (Valenciennes, 1839) and *R. porosus* (Poey, 1861), family Carcharhinidae. The ‘parallel dentition arrangement’ (Figure 8f) is characterized by teeth aligned parallel to adjacent teeth, without overlapping or alternation. This represents the first record of parallel dentition arrangement within the order Carcharhiniformes.

### 3.1 Dental morphology within Carcharhiniformes

In this section, we provide information on tooth morphology divided by each carcharhiniform family, except Dichichthyidae. A summary of the main dental features by family is presented in Table 2.

**TABLE 2:** Dental characters and their alternate states by family.

Families	Number of cusps	Striae	Ectodermal pits	Basal groove	Serrations	Limiting zone
Atelomycteridae	1–9	0–20 (upper) 0–16 (lower)	Present	Present	Absent	Absent
Carcharhinidae	1	Mostly absent	Rarely present	Mostly absent	Present	Present
Galeocerdonidae	1–5	Absent	Absent	Absent	Present	Present
Hemigaleidae	0–7	Mostly absent	Absent	Present	Present in *Hemipristis*	Absent
Leptochariidae	3	Present	Mostly absent	Present	Absent	Absent
Pentanchidae	1–9	1–15	Present	Present	Absent	Absent
Proscylliidae	1–5	7–11	Present	Present	Absent	Absent
Pseudotriakidae	5–7	12–21	Present	Present	Absent	Absent
Scyliorhinidae	1–5	1–33	Present	Present	Absent	Absent
Sphyrnidae	1	Absent	Absent	Absent	Present in some tooth groups	Present in some tooth groups
Triakidae	0–5	1–25 (upper teeth) 1–18 (lower teeth)	Present	Present	Absent	Absent

#### 3.1.1 Atelomycteridae (Figure 9—*Atelomycterus fasciatus*, Compagno & Stevens, 1993)

**FIGURE 9:**
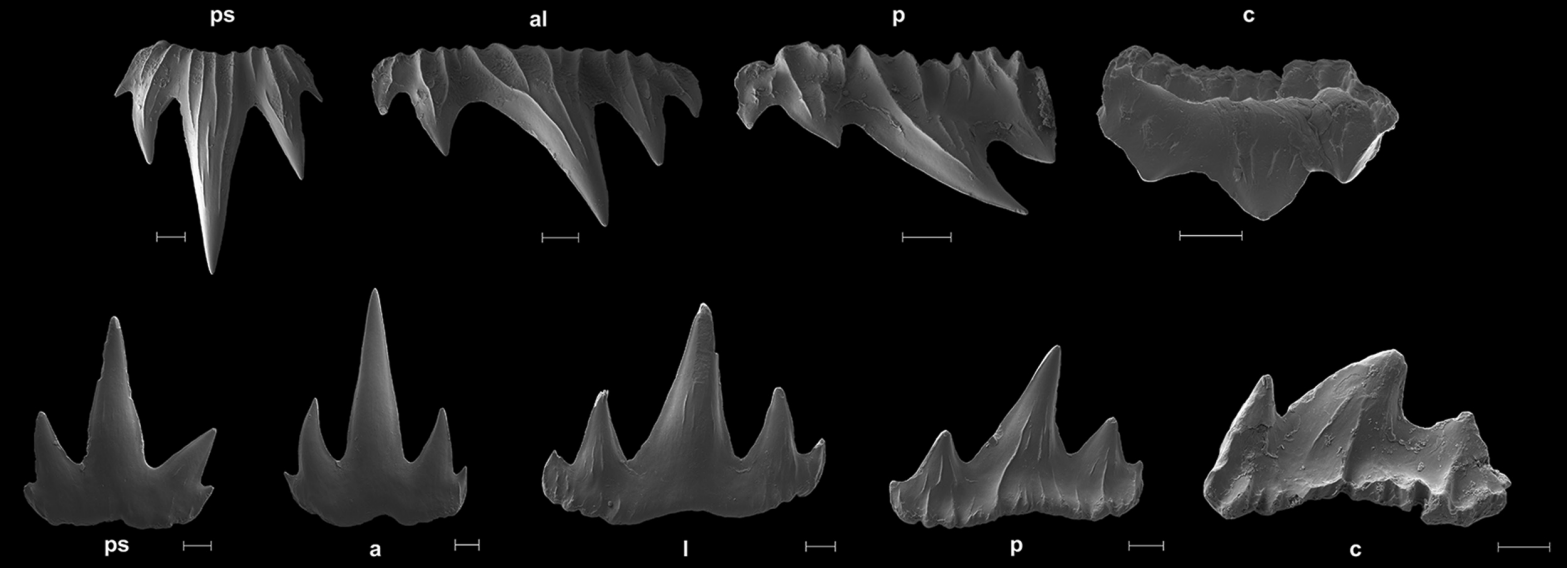
Labial face of teeth of a female specimen of *Atelomycterus fasciatus*. MZUSP 118095, 363 mm TL. Scale bars = 100 μm. a, anterior; al, anterolateral; c, commissural; l, lateral; p, posterior; ps, parasymphyseal.

Teeth predominantly multicuspid, with the number of cusps ranging from one to nine and increasing toward the commissure. Principal cusp of posterior and commissural teeth mostly oblique, oriented toward the commissure. Crown base frequently concave with protuberances usually present. Striae generally present, ranging from zero to 20 on upper teeth and from zero to 16 on lower teeth, often exceeding half the height of the principal cusp. Ectodermal pits typically present and restricted to the crown base. Basal groove present in all examined species. Serrations and a limiting zone are absent.

#### 3.1.2 Carcharhinidae (Figure 10—*Carcharhinus signatus*, Poey, 1868)

**FIGURE 10:**
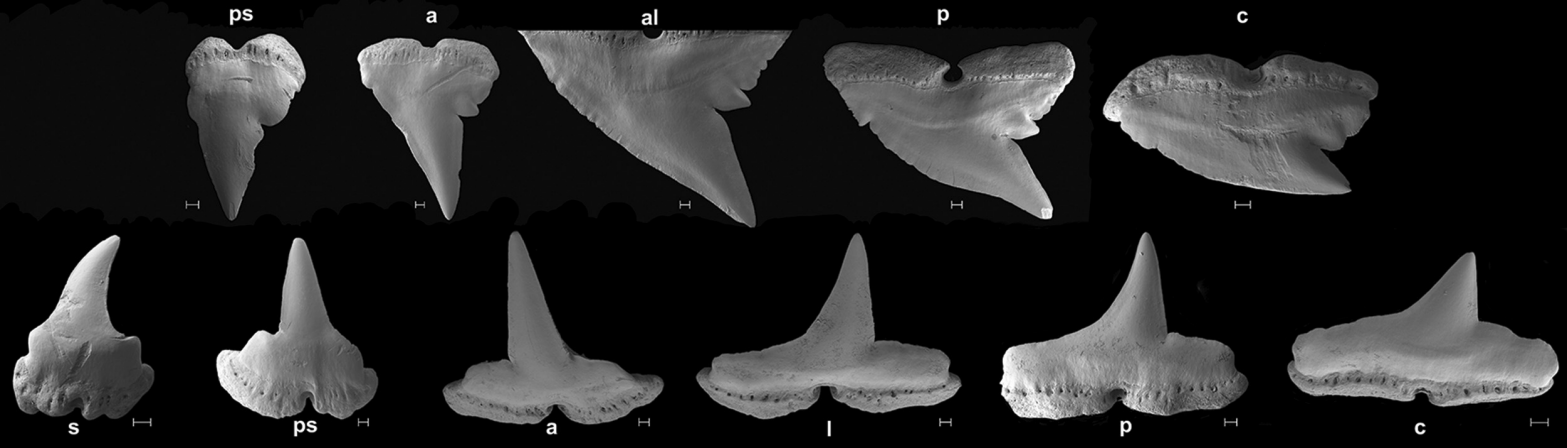
Labial face of teeth of a female specimen of *Carcharhinus signatus*. UERJ 2023, no TL data. Scale bars = 200 μm. a, anterior; al, anterolateral; c, commissural; l, lateral; p, posterior; ps, parasymphyseal; s, symphyseal.

Teeth predominantly wider than high and unicuspid, with a distal lateral expansion or a lateral expansion on both sides of the principal cusp. Obliquity of the principal cusp increases toward the commissure. Serrations are more frequently observed in the genus *Carcharhinus*, but may also be present in *Rhizoprionodon*. Protuberances at the crown base are absent. Crown base is generally straight. Striae are mostly absent, and when present, restricted to commissural and/or posterior teeth, not exceeding half the height of the principal cusp. Basal groove is generally absent. Ectodermal pits are rarely present. Limiting zone is commonly present.

#### 3.1.3 Galeocerdonidae (Figure 11—*Galeocerdo cuvier*, Péron & Lesueur, 1822)

**FIGURE 11:**
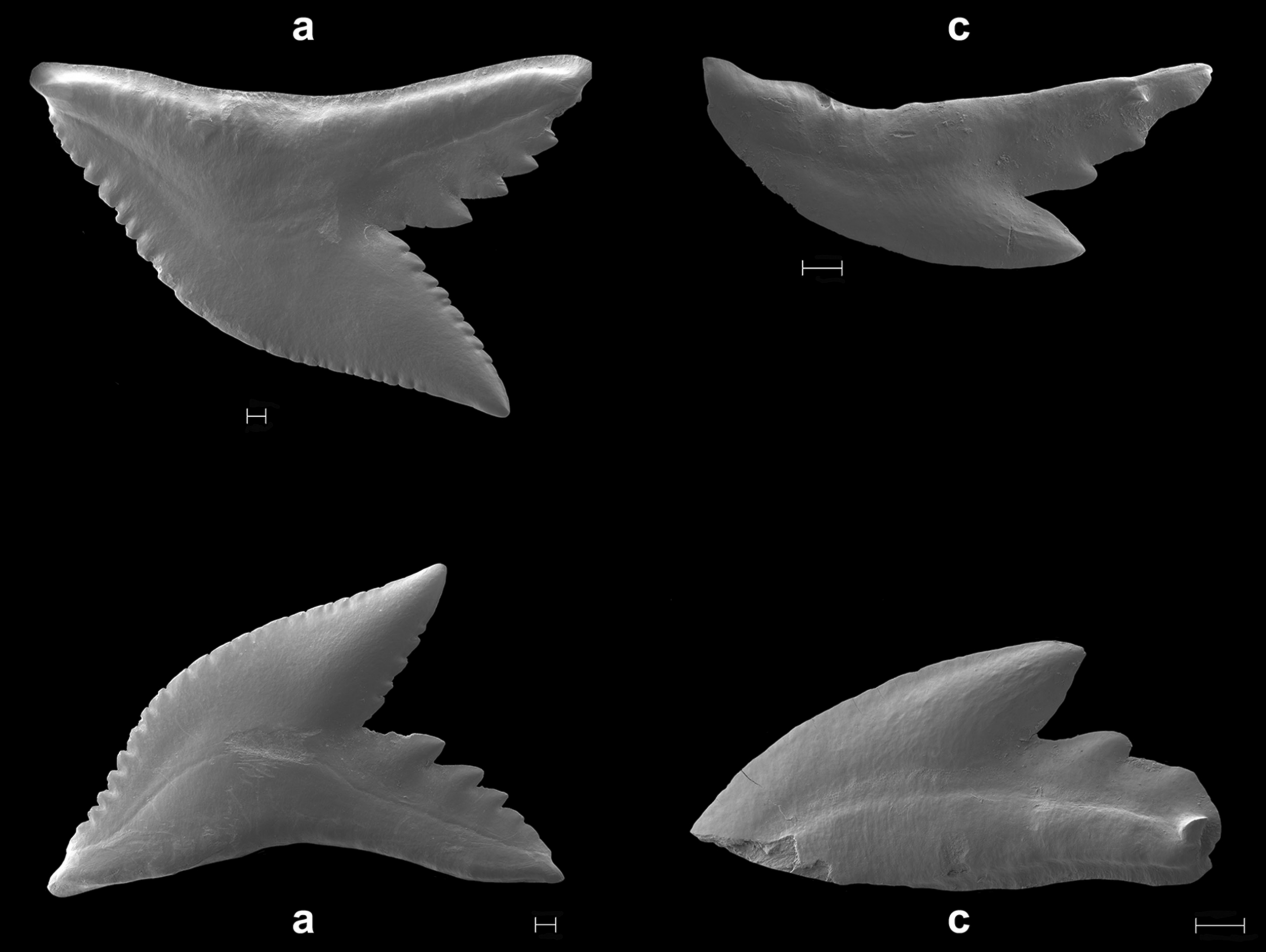
Labial face of teeth of a female specimen of *Galeocerdo cuvier*.MZUSP 112027, no TL data. Scale bars = 200μ m. a, anterior; c, commissural.

In juvenile specimens, teeth are multicuspid with one to five cusps. The principal cusp is strongly oblique toward the commissure, except in the symphyseal tooth. The crown base varies from straight to slightly concave. Serrations are present in the mesial and distal edges. A limiting zone is present in some tooth groups. Striae, protuberances at the crown base, and ectodermal pits are absent.

In adults, the teeth are monocupid with fine serrations. Macdonald and Barron (1868), Fowler (1941) and Bigelow and Schroeder (1948) described secondary serrations occurring along the edges of the coarse serrations. However, secondary serrations were not observed in the present study. The distinction between serrations and accessory cusps in *Galeocerdo cuvier* is ambiguous, and the boundary between them can be arbitrary. Here, accessory cusps were differentiated from serrations based on their more triangular apices (vs. more rounded in serrations) and their greater degree of development, since serrations are comparatively less pronounced.

#### 3.1.4 Hemigaleidae (Figure 7—*Paragaleus pectoralis*, Garman, 1906)

From symphyseal to anterior teeth higher than wide, posterior and commissural teeth wider than high, generally multicuspid mainly in the upper jaw, with zero to seven cusps in the upper teeth row and zero to three in the lower. Obliquity of the principal cusp increases toward the commissure. Crown base concave. Basal groove generally present. Striae mostly absent; when present, restricted to posterior and commissural teeth. Protuberances at the crown base, ectodermal pits, limiting zone, and serrations only present in *Hemipristis elongata* (Klunzinger, 1871).

#### 3.1.5 Leptochariidae (Figure 6—*Leptocharias smithii*, Müller & Henle, 1839)

Teeth higher than wide, multicuspid usually with three cusps. Principal cusp erect or slightly oblique toward the commissure. Crown base frequently concave. Protuberances in the crown base only in the symphyseal and parasymphyseal. Striae frequently present and more prominent in the lower teeth. Basal groove present, except at the lateral teeth and lower posteriors. Ectodermal pits absent, except in the lower posterior teeth. Limiting zone and serrations absent.

#### 3.1.6 Pentanchidae (Figure 12—*Galeus melastomus*, Rafinesque, 1810)

**FIGURE 12:**
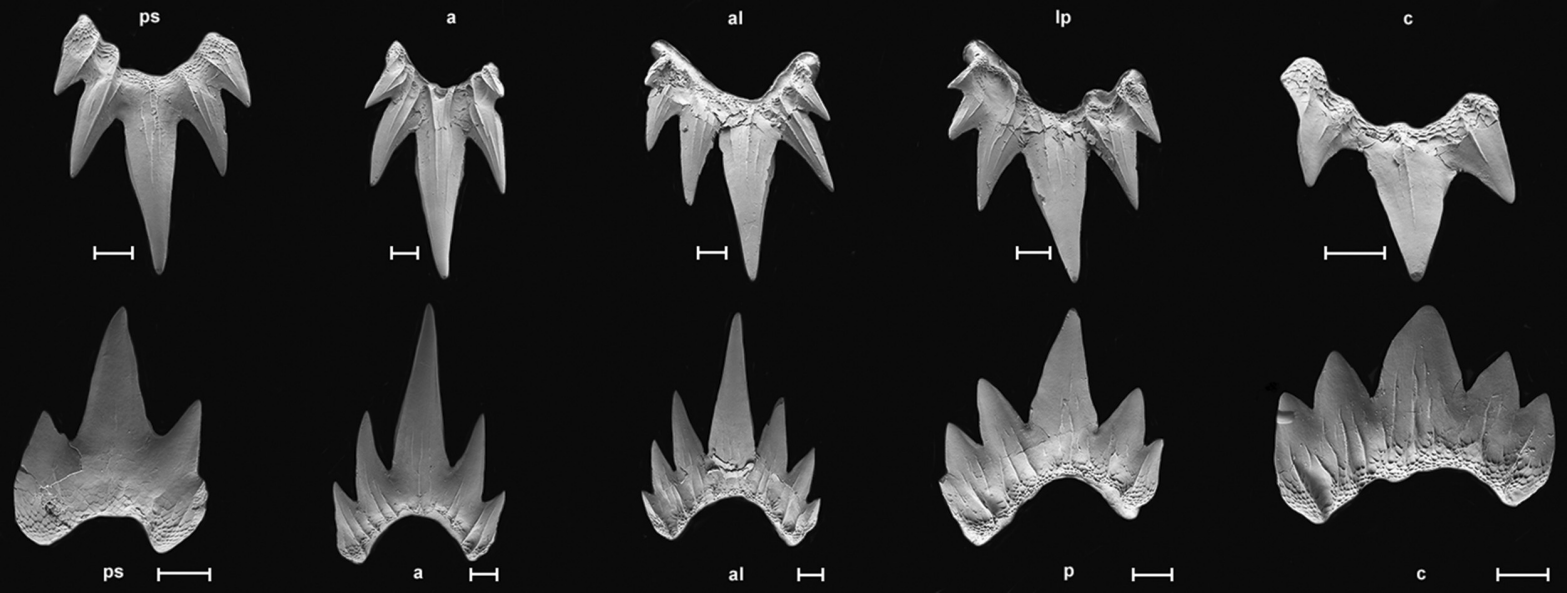
Labial face of teeth of a female specimen of *Galeus melastomus*. USP not cataloged, 370 mm TL. Scale bars = 100 μm. a, anterior; al, anterolateral; c, commissural; lp, lateroposterior; p, posterior; ps, parasymphyseal.

Teeth generally multicuspid, with one to nine cusps. Striae mostly present, ranging from one to 15 and often extending beyond half the height of the principal cusp. Crown base typically concave. Ectodermal pits frequently present and usually restricted to the crown base. Basal groove present, except in *Apristurus* species and *Galeus polli*. Protuberances at the crown base generally present. Serrations and limiting zone are absent.

#### 3.1.7 Proscylliidae (Figure 13—*Eridacnis barbouri*, Bigelow & Schroeder, 1944)

**FIGURE 13:**
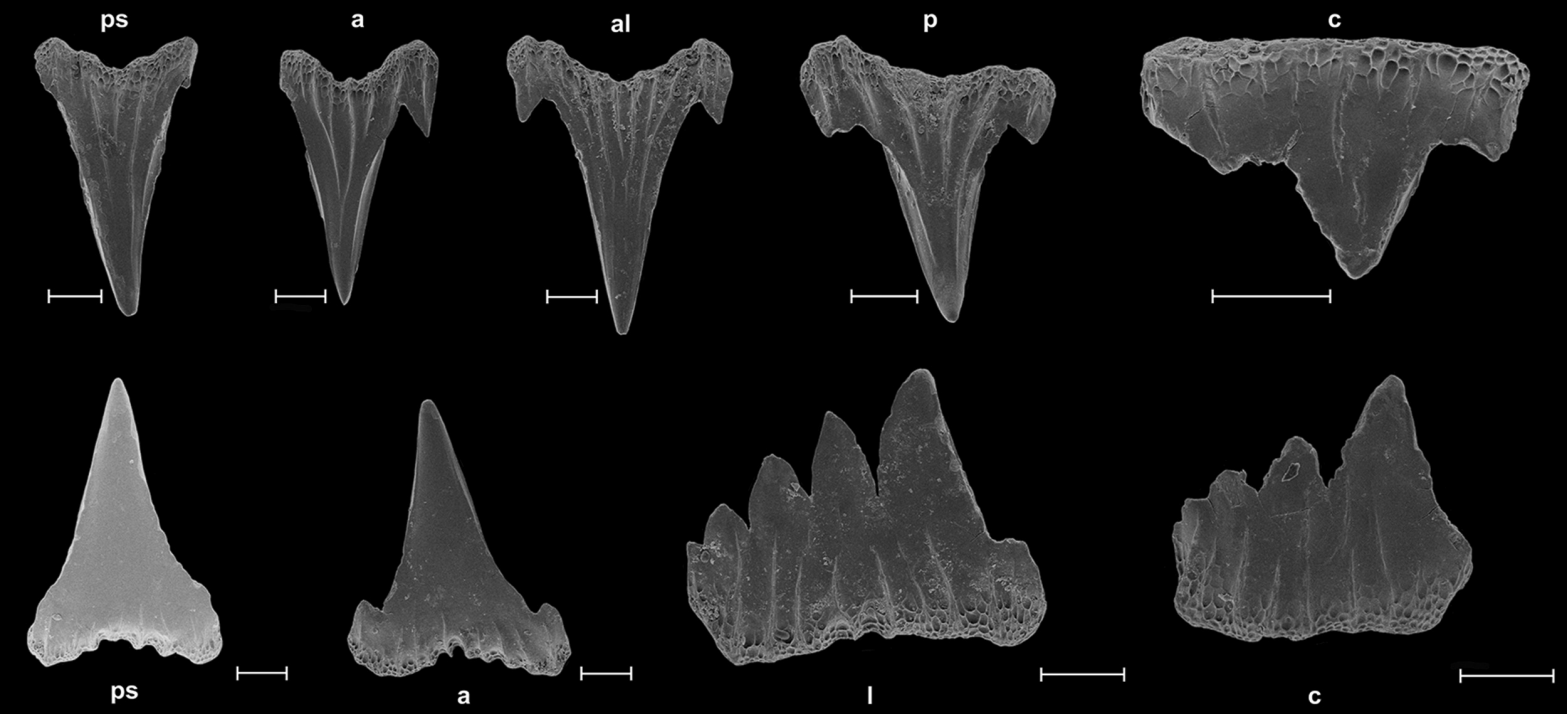
Labial face of teeth of a male specimen of *Eridacnis barbouri*. USNM 188023, 265 mm TL. Scale bars = 100 μm. a, anterior; al, anterolateral; c, commissural; l, lateral; p, posterior; ps, parasymphyseal.

Usually, teeth are higher than wide from parasymphyseal to anterolateral and wider than high from lateral to commissural, generally multicuspid, with one to five cusps. The number of cusps and the obliquity of the principal cusp increase toward the commissure. The crown base is usually concave. The basal groove is generally present. Striae are present, ranging from seven to 11. Ectodermal pits are present and restricted to the crown base. The basal groove and protuberances at the crown base are mostly present. The limiting zone and serrations are absent.

#### 3.1.8 Pseudotriakidae (Figure 14—*Gollum attenuatus*, Garrick, 1954)

**FIGURE 14:**
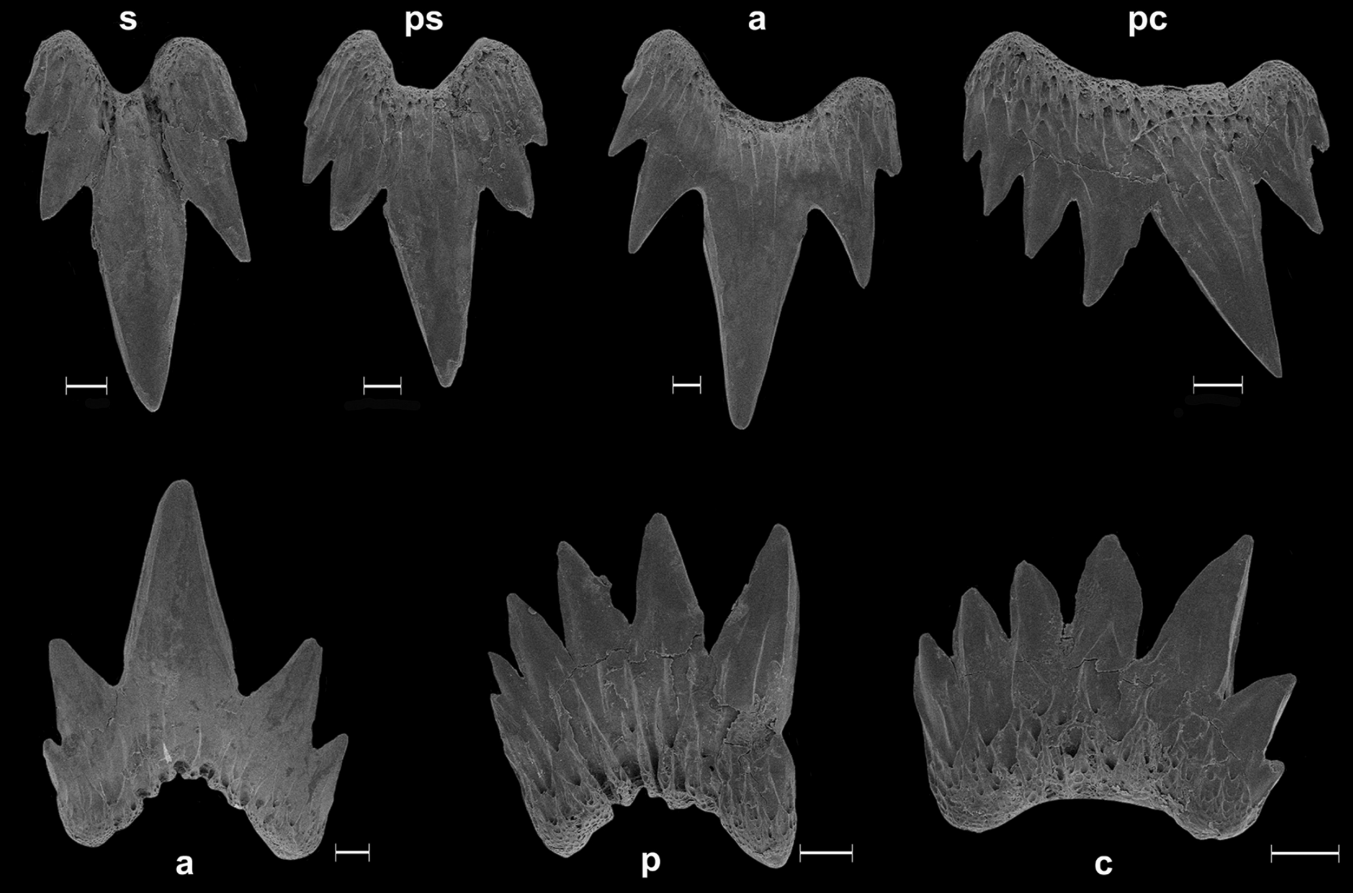
Labial face of teeth of a female specimen of *Gollum attenuatus*. NSMT 42855, 950 mm TL. Scale bars = 100 μm. a, anterior; c, commissural; pc, posterocommissural; ps, parasymphyseal; s, symphyseal.

Teeth higher than wide and multicuspid, with five to seven cusps. The number of cusps and the obliquity of the principal cusp increase toward the commissure. Crown base concave. Basal groove generally present. Striae present, ranging from 12 to 21 in the upper teeth and from five to 13 in the lower; varying from restricted to the crown base to extending beyond the principal cusp height. Ectodermal pits present and restricted to the crown base. Protuberances at the crown base are mostly absent. Limiting zone and serrations are absent.

#### 3.1.9 Scyliorhinidae (Figure 15—*Scyliorhinus ugoi* Soares et al., 2015)

**FIGURE 15:**
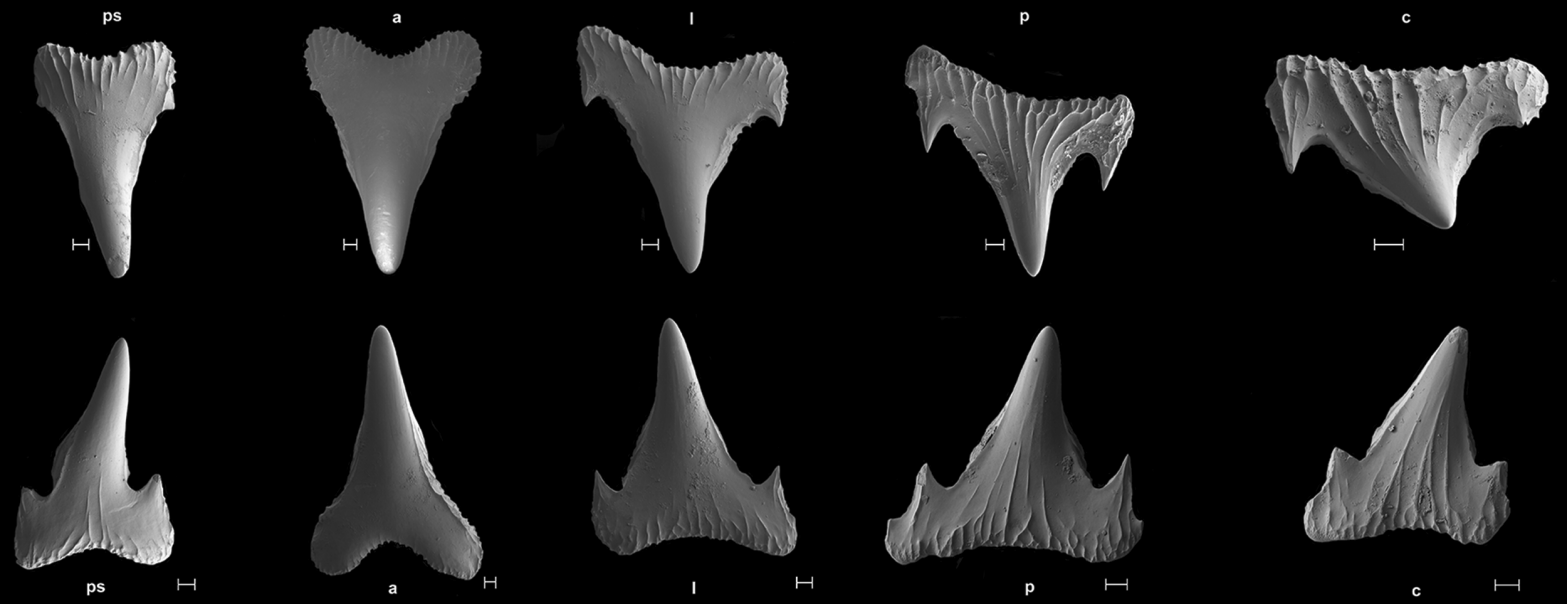
Labial face of teeth of a male specimen of *Scyliorhinus ugoi*. USP not cataloged, 525 mm TL. Scale bars = 100 μm. a, anterior; c, commissural; l, lateral; p, posterior; ps, parasymphyseal.

Teeth predominantly multicuspid, with one to five cusps. Obliquity of the principal cusp and the concavity of the crown base increase toward the commissure. Striae are mostly present, ranging from one to 33. Ectodermal pits are generally present and mostly restricted to the crown base. Basal groove is generally present. Protuberances at the crown base are commonly present. Serrations and limiting zone are absent.

#### 3.1.10 Sphyrnidae (Figure 5—*Sphyrna lewini*, Griffith & Smith, 1834)

Teeth wider than high and generally monocuspid. Obliquity of the principal cusp increases toward the commissure. Striae, ectodermal pits, basal groove, and protuberances at the crown base are absent. Limiting zone and serrations may be present in some tooth groups.

#### 3.1.11 Triakidae (Figures 16 and 17—*Galeorhinus galeus*, Linnaeus, 1758 and *Mustelus schmitti* Springer, 1939)

**FIGURE 16:**
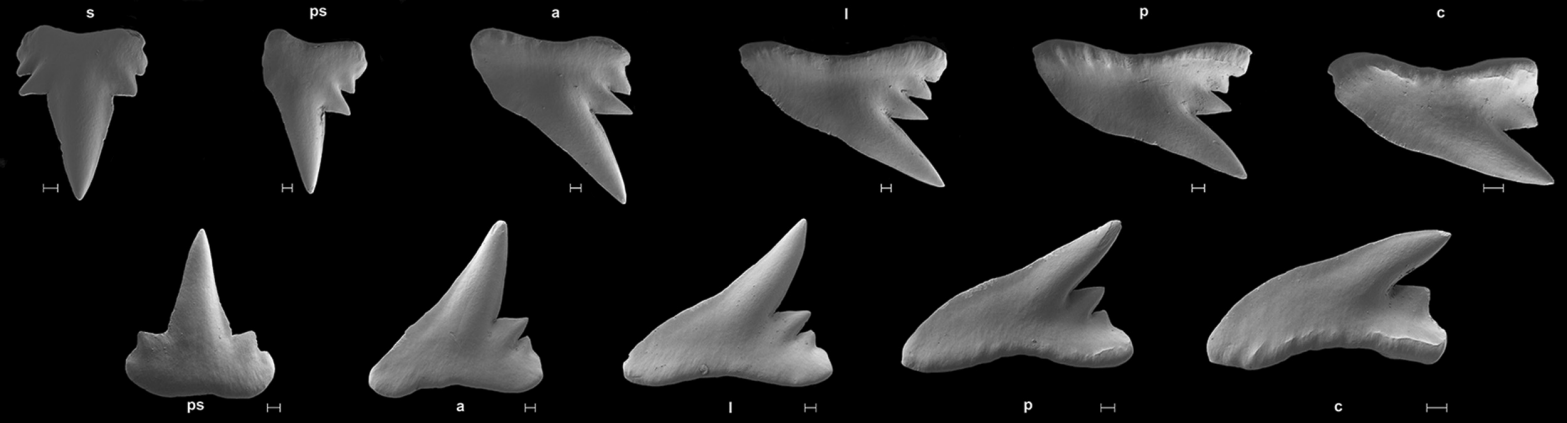
Labial face of teeth of a male specimen of *Galeorhinus galeus*. UERJ 291, 498 mm TL. Scale bars = 100 μm. a, anterior; c, commissural; l, lateral; p, posterior; ps, parasymphyseal; s, symphyseal.

**FIGURE 17:**
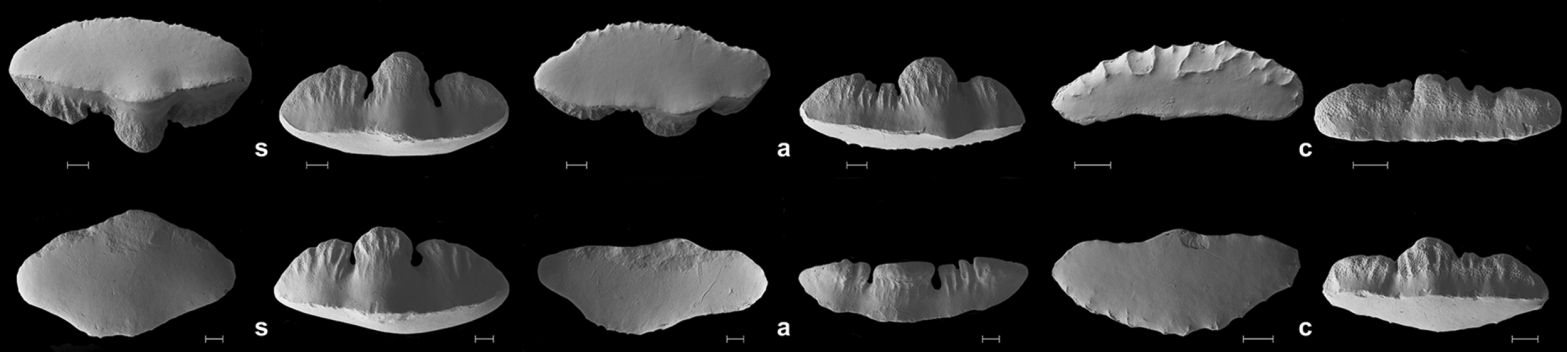
Occlusal and labial face of teeth of a female specimen of *Mustelus schmitti*. MZUSP not cataloged, 620 mm TL. Scale bars = 100 μm. a, anterior; c, commissural; s, symphyseal.

Teeth are generally multicuspid, with zero to five cusps. The number of cusps decreases, while the obliquity of the principal cusp increases toward the commissure. Striae are frequently present, ranging from one to 25 in the upper teeth and from one to 18 only in the commissural teeth of the lower jaw. Ectodermal pits and protuberances at the crown base are variable. Basal groove is usually present. Serrations and limiting zone are absent.

### 3.2 Heterondonty and arrangements

In Carcharhiniformes, tooth arrangements vary between the upper and lower jaws and among different regions within the same jaw. Typically, mesial tooth groups exhibit a distinct arrangement compared to posterior and commissural teeth. This section describes family‐level patterns of tooth heterodonty and arrangements, including their defining characteristics and specific examples. A summary of heterodonty types observed by family and each species examined is provided in Table 3, and detailed below.

**TABLE 3:** Heterodonty (dignathic, gynandric, monognathic, and ontogenetic) observed in the examined species within Carcharhiniformes.

Taxa	Dignathic heterodonty	Gynandric heterodonty	Monognathic heterodonty	Ontogenetic heterodonty	References
Atelomycteridae					
*Atelomycterus fasciatus*	Present	Absent	Present	–	Present study; Compagno & Stevens, 1993
*Schroederichthys bivius*	Present	Present	Present	–	Present study; Springer, 1979; Compagno, 1984, 1988
*Schroederichthys chilensis*	Present	Present	Present	–	Present study; Kato et al., 1967; Springer, 1979; Soto, 2001b
*Schroederichthys maculatus*	Present	Absent	Present	–	Present study; Springer, 1979
*Schroederichthys saurisqualus*	Present	Present	Present	–	Present study; Soto, 2001b; Pagnoncelli, 2009
Carcharhinidae					
*Carcharhinus brevipinna*	Present	–	Present	–	Present study
*Carcharhinus* cf. *falciformis*	Present	Present	Present	–	Present study; Müller & Henle (1838–1841); Poey (1861); Bigelow and Schroeder (1948)
*Carcharhinus plumbeus*	Present	–	Present	–	personal observations; Garrick, 1982
*Carcharhinus signatus*	Present	–	Present	Present	Present study; Raschi et al., 1982; Compagno, 1984; Garrick, 1985
*Negaprion brevirostris*	Present	–	Present	Present	Present study; Compagno, 1984; Purdy, 2006
*Rhizoprionodon lalandii*	Present	Present	Present	Present	Present study; Springer, 1964; Cadenat & Blanche, 1981; Compagno, 1984; Gomes, 1988
*Rhizoprionodon porosus*	Present	Absent	Present	Present	Present study; Poey, 1861; Springer, 1964; Cadenat & Blanche, 1981; Compagno, 1984, 2002; Gomes, 1988
Galeocerdonidae					
*Galeocerdo cuvier*	Present	Absent	Present	Present	Present study; Compagno, 1988
Hemigaleidae					
*Paragaleus pectoralis*	Present	–	Present	–	Present study; Herman et al., 1991
Leptochariidae					
*Leptocharias smithii*	Present	Present	Present	–	Present study; Compagno, 1988; Cadenat & Blanche, 1981; Herman et al., 1991
Pentanchidae					
*Apristurus australis*	Present	Present	Present	–	Present study; Sato et al., 2008
*Apristurus laurussonii*	Present	Present	Present	–	Present study; Springer, 1979, Herman et al., 1990; Iglésias & Nakaya, 2004
*Apristurus parvipinnis*	Present	–	Present	–	Present study; Springer, 1979; Compagno, 1984
*Bythaelurus lutarius*	Absent	Present	Present	–	Present study; Springer, 1979; Bass et al., 1975
*Cephalurus* sp.	Present	–	Present	–	Present study; Herman et al., 1990
*Figaro boarmani*	Present	–	Present	–	Present study
*Galeus antillensis*	Present	Present	Present	–	Present study
*Galeus melastomus*	Present	Present	Present	–	Present study; Herman et al., 1990
*Galeus mincaronei*	Present	Present	Present	–	Present study; Soto, 2001a
*Galeus polli*	Present	–	Present	–	Present study
*Halaelurus natalensis*	–	Absent	Present	–	Present study; Bass et al., 1975
*Haploblepharus edwardsii*	Present	Present	Present	–	Present study; Bass et al., 1975; Herman et al., 1990
*Holohalaelurus regani*	–	–	Present	–	Present study; Bass et al., 1975; Springer, 1979; Herman et al., 1990
*Holohalaelurus* sp.	Present	–	Present	–	Present study
Proscylliidae					
*Eridacnis barbouri*	Present	–	Present	–	Present study
Pseudotriakidae					
*Gollum attenuatus*	Present	–	Present	–	Present study; Compagno, 1988; Herman et al., 1991
Scyliorhinidae					
*Cephaloscyllium sufflans*	–	–	Present	–	Present study; Fowler, 1941; Bass et al., 1975; Bass et al., 1986
*Cephaloscyllium umbratile*	Present	–	Present	–	Present study
*Poroderma africanum*	Present	–	Present	–	Present study; Müller & Henle, 1838–1841; Smith, 1845; Fowler, 1925, 1941; Bass et al., 1975; Heemstra & Heemstra, 2004
*Poroderma pantherinum*	–	–	Present	–	Present study; Bass et al., 1975; Herman et al., 1990
*Scyliorhinus cabofriensis*	Present	Present	Present	–	Present study; Soares et al., 2016
*Scyliorhinus capensis*	Present	Present	Present	Present	Present study; Bass et al., 1975; Soares & de Carvalho, 2019
*Scyliorhinus ugoi*	Present	Present	Present	–	Present study; Soares et al., 2016
Sphyrnidae					
*Sphyrna lewini*	Present	Present	Present	Present	Present study; Bigelow & Schroeder, 1948
Triakidae					
*Galeorhinus galeus*	Present	–	Present	Present	Present study; Jordan & Gilbert, 1882; Herman et al., 1988
*Hemitriakis japanica*	Present	Present	Present	Present	Present study; Compagno, 1970, 1988; Herman et al., 1988
*Iago omanensis*	Present	Present	Present	Absent	Present study; Compagno & Springer, 1971
*Mustelus canis*	Present	–	Present	Present	Present study; Heemstra, 1997; Compagno, 1984; Rosa & Gadig, 2010
*Mustelus schimitti*	Present	Present	Present	–	Present study; Springer, 1939; Rosa & Gadig, 2010; Belleggia et al., 2014
*Triakis semifasciata*	Present	–	Present	Present	Present study; Compagno, 1970; Herman et al., 1988

*Note*: –, data not available. Additional references are also presented.

Atelomycteridae, Pentanchidae, Pseudotriakidae, and Scyliorhinidae: moderate to strong monognathic heterodonty and generally weak dignathic heterodonty. The independent sub‐arrangement is predominant, except in *Figaro boardmani* (Whitley, 1928) and *Galeus polli* Cadenat, 1959 (Pentanchidae), as well as in the family Pseudotriakidae, which exhibits an imbricate sub‐arrangement in posterior and commissural tooth groups.

Carcharhinidae: strong dignathic heterodonty in *Carcharhinus*, whereas monognathic heterodonty is weak. The alternate dentition in the upper jaw typically exhibited overlapping sub‐arrangements, but representatives of the genus also display independent or imbricate sub‐arrangements. The genus *Rhizoprionodon* exhibited weak monognathic and dignathic heterodonty. Its dental arrangement is generally a mixed sub‐arrangement, with imbricate sub‐arrangement observed in the posterior and commissural teeth of males. Lastly, *Negaprion brevirostris* (Poey, 1868) presents moderate monognathic heterodonty and weak dignathic heterodonty, with an independent sub‐arrangement.

Galeocerdonidae and Sphyrnidae: monognathic and dignathic heterodonty weak. *Galeocerdo cuvier* (Galeocerdonidae) exhibits the independent sub‐arrangement. *Sphyrna lewini* (Griffith & Smith, 1834) exhibits imbricate sub‐arrangement; in the lower jaw of females, the dental arrangement was an independent sub‐arrangement from symphyseal to anterolateral teeth and imbricate in posterior and commissural teeth.

Hemigaleidae, Leptochariidae, and Proscylliidae: moderate to strong monognathic heterodonty and strong dignathic heterodonty. Hemigaleidae presents a mixed sub‐arrangement, and Leptochariidae an independent sub‐arrangement. In Proscylliidae, independent sub‐arrangement is observed in the upper jaw and from parasymphyseal to lateral teeth in the lower jaw, with overlap sub‐arrangement in the posterior and commissural lower teeth.

Triakidae: monognathic heterodonty ranged from weak to moderate, and dignathic heterodonty is weak. Within this family, the sub‐arrangements are significantly varied. *Galeorhinus galeus* (Linnaeus, 1758) and *Iago omanensis* Norman, 1939 present a mixed sub‐arrangement. *Hemitriakis japanica* (Müller & Henle, 1839) has an overlapping sub‐arrangement, except in the commissural lower teeth, which exhibit an independent sub‐arrangement. *Triakis semifasciata* Girard, 1855 presents an independent sub‐arrangement from parasymphyseal to lateral teeth in the upper jaw and from symphyseal to anterior teeth in the lower jaw, with an imbricate sub‐arrangement in the posterior and commissural teeth of the upper jaw and from lateral to commissural teeth in the lower jaw. *Mustelus* is the only genus to present the pavement dentition in the order Carcharhiniformes.

## 4 DISCUSSION

The dental crown variation observed within the order Carcharhiniformes is broad and complex, primarily driven by monognathic, dignathic, ontogenetic, and gynandric heterodonty (Garrick, 1982, 1985; Compagno, 1984, 1988; Gomes, 1988; Herman et al., 1991; Soares & de Carvalho, 2019; Soares & Zanini, 2023). Consequently, inconsistent or superficial dental descriptions, often limited to anterior teeth, are commonly presented in the literature. However, a comprehensive understanding of heterodonty is essential to elucidate both the extant diversity and evolutionary history of the group (Guinot et al., 2018; Mollen, 2019).

Compagno (1979) previously identified strong monognathic and dignathic heterodonty as derived features in Carcharhinidae, Galeocerdonidae, and Sphyrnidae. According to our results, strong monognathic heterodonty was not observed in these taxa, nor was strong dignathic heterodonty observed in Galeocerdonidae, Sphyrnidae, or *Rhizoprionodon* (Carcharhinidae). Catsharks (Atelomycteridae, Pentanchidae, Scyliorhinidae) typically exhibit gynandric heterodonty, but no information is available regarding dental variation during their ontogeny. In Carcharhinidae and Triakidae, the occurrence of ontogenetic heterodonty is more frequent than gynandric heterodonty. The family Sphyrnidae exhibits gynandric and ontogenetic heterodonty, while Leptochariidae shows gynandric heterodonty but lacks information on ontogenetic heterodonty. Conversely, Galeocerdonidae does not exhibit gynandric heterodonty, and its ontogenetic heterodonty is weak. The dentition of *Galeocerdo cuvier* (Péron & Lesueur, 1822) is well documented, and one of its most distinctive features is the presence of secondary serrations (Moyer & Bemis, 2017; Türtscher et al., 2021). However, secondary serrations were observed in the juvenile individual examined in this study. Although the dentition of the tiger shark has been extensively described, this finding highlights the need for further research on heterodonty in the species.

Types of heterodonty were first documented for *Carcharhinus brevipinna* (Valenciennes, 1839) (Carcharhinidae), *Cephaloscyllium umbratile* Jordan & Fowler, 1903 (Figure 18), and *Scyliorhinus capensis* (Müller & Henle, 1838) (Figure 4) (Scyliorhinidae), *Eridacnis barbouri* (Bigelow & Schroeder, 1944) (Proscylliidae) (Figure 13), *Galeorhinus galeus* (Linnaeus, 1758) (Triakidae) (Figure 16), *Figaro boardmani* (Whitley, 1328), *Galeus antillensis* Springer, 1979 (Figure 19), and *G. polli* Cadenat, 1959 (Figure 20) (Pentanchidae) (see Table 3).

**FIGURE 18:**
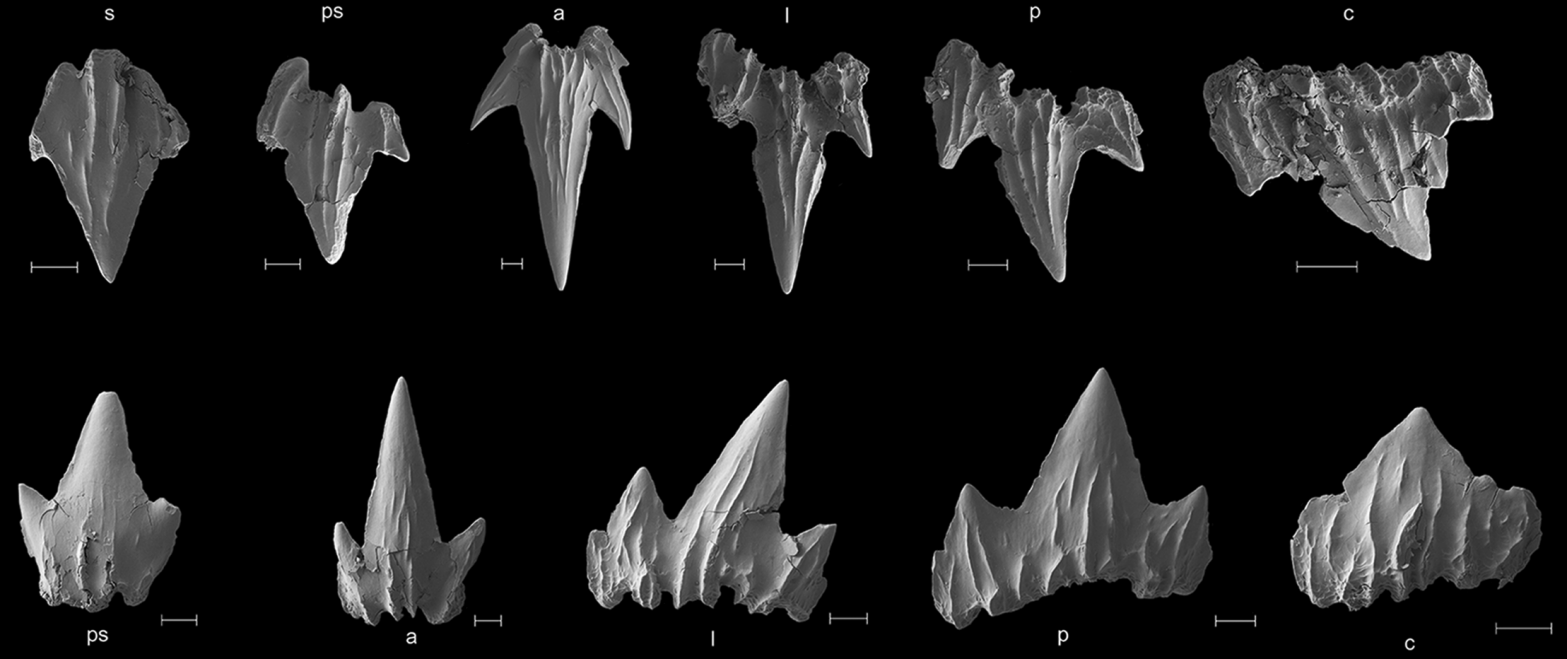
Labial face of teeth of a male specimen of *Cephaloscyllium umbratile*. USP not cataloged, 409 mm TL. Scale bars = 100 μm. a, anterior; c, commissural; l, lateral; p, posterior; ps, parasymphyseal; s, symphyseal.

**FIGURE 19:**
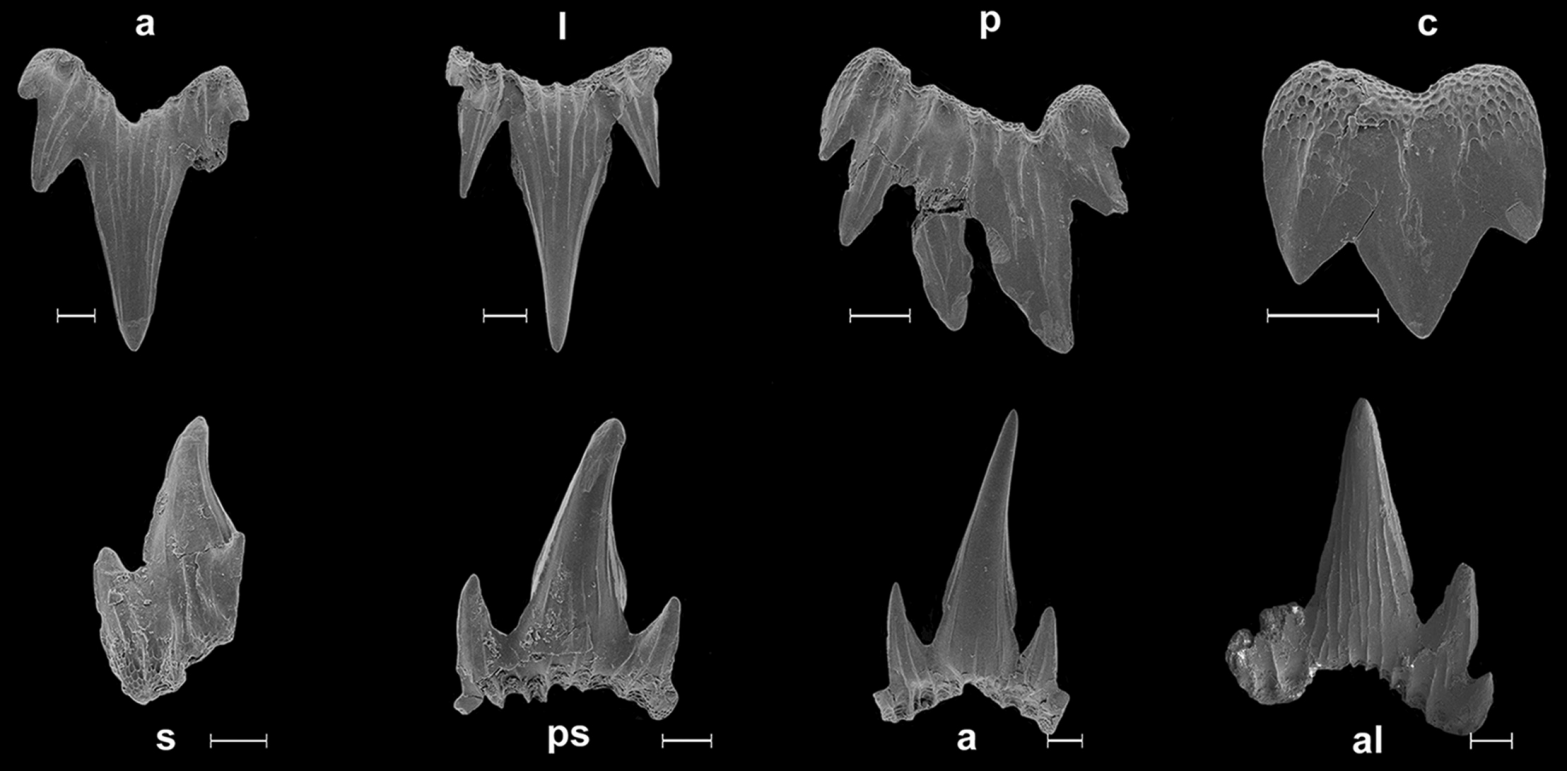
Labial face of teeth of a specimen of *Galeus antillensis*. UF 14824. Scale bars = 100 μm. a, anterior; al, anterolateral; c, commissural; l, lateral; p, posterior; ps, parasymphyseal; s, symphyseal.

**FIGURE 20:**
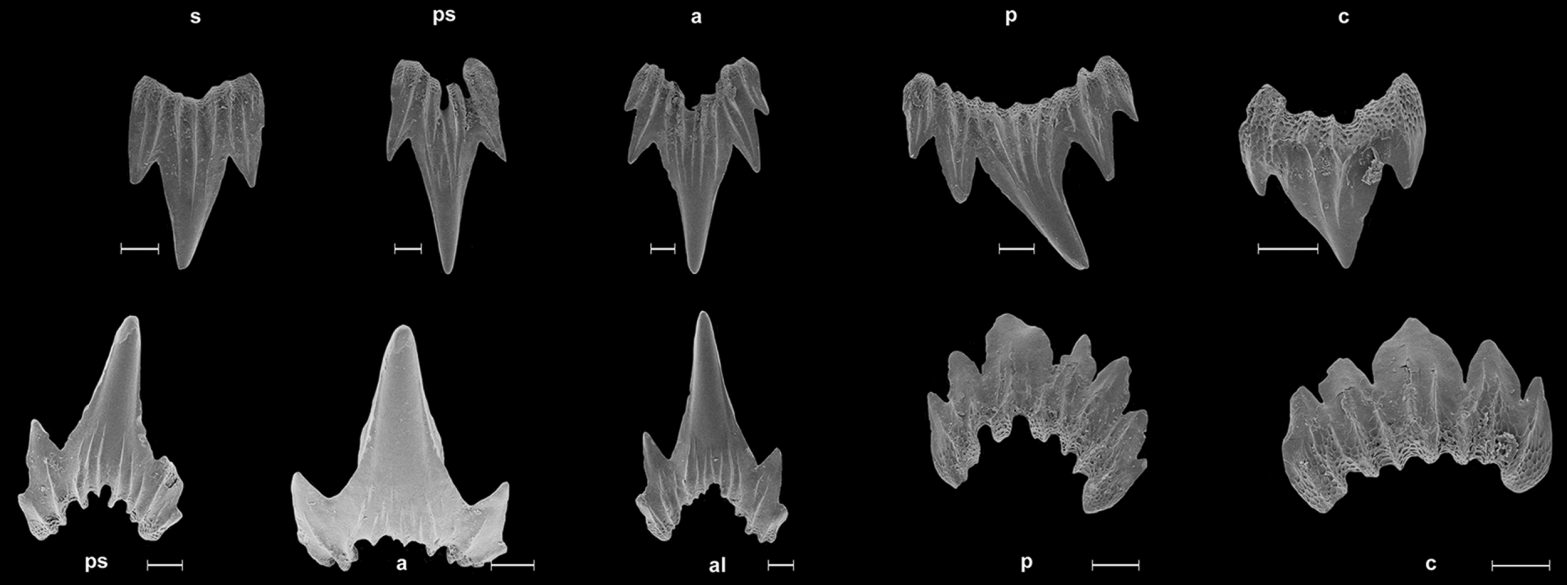
Labial face of teeth of a male specimen of *Galeus polli*. USNM 221610, 378 mm TL. Scale bars = 100 μm. a, anterior; al, anterolateral; c, commissural; p, posterior; ps, parasymphyseal; s, symphyseal.

The observation of ontogenetic heterodonty in *Scyliorhinus capensis* (Figure 4) suggests that ontogenetic dental differences may exist within the group, although they remain understudied and poorly documented. Representatives of the family Etmopteridae (order Squaliformes) possess upper teeth resembling those of Scyliorhinidae and Pentanchidae. However, in Etmopteridae, the teeth are more labiolingually flattened and lack striae and ectodermal pits. The dentition of Lamniformes, the sister group to Carcharhiniformes, is somewhat similar to that of Carcharhinidae but differs in aspects such as weaker dignathic heterodonty, accessory cusps that are more common, and serrations that are less frequently observed than in Carcharhinidae.

Typically, sexual differences in dentition involve variation in the number of cusps and tooth size. Whether males or females exhibit a greater number of cusps or larger teeth varies by species, and no consistent pattern was observed. Since sharks exhibit mating and copulatory behavior that involves biting and oral grasping, the sexual differences observed in their dentition could be related to reproduction (Carrier et al., 2004). Heterodonty and tooth arrangements could also be correlated with feeding strategies and ecological niches. For example, the independent sub‐arrangement in *Apristurus* and *Scyliorhinus* is associated with high levels of both monognathic and dignathic heterodonty, possibly reflecting an increased capacity for processing varied prey types. The pavement dentition in *Mustelus*, by contrast, supports a more uniform dentition, consistent with a durophagous feeding mode (Compagno, 1984; Gadig, 2001; Cappetta, 2012).

Although the works of Herman et al. (1988, 1990, 1991) provided detailed morphological descriptions of shark dentitions, the present study expands the knowledge on heterodonty and tooth arrangements at the family level. Furthermore, the definition and consistent application of five sub‐arrangements of dentition, including the newly proposed “parallel dentition” for Carcharhiniformes, offer a novel and operational framework not formalized in earlier literature. This comparative synthesis builds upon the taxonomic reference model proposed by Cappetta (2012), adding high‐resolution data from extant species to enhance fossil interpretations.

This study highlights the diagnostic value of heterodonty and tooth arrangement at both species and family levels. High‐resolution SEM imaging enabled the identification of key morphological features, such as striae length and number, the presence and position of ectodermal pits, and basal ornamentation that are often overlooked in traditional descriptions. These features are critical for distinguishing closely related taxa and correcting historical taxonomic misassignments. Our findings could potentially impact paleontological research since fossil shark identification frequently relies on isolated teeth, and a detailed understanding of heterodonty in extant taxa is essential for distinguishing intraspecific variation from differences between species.

## 5 CONCLUSION

The present study demonstrates that detailed analyses of heterodonty supported by high‐resolution imaging are essential for refining taxonomic classifications within Carcharhiniformes. Since the dentition of extant taxa forms the basis for identifying and classifying extinct species, and because many large‐bodied species are more commonly found and preserved in juvenile stages, it is critical to document all forms of heterodonty (monognathic, dignathic, gynandric, and ontogenetic) to minimize misidentifications. The identification of subtle morphological traits not only aids in distinguishing closely related taxa but also helps correct historical taxonomic inaccuracies. These findings highlight the need to integrate modern imaging techniques into taxonomic research, as they enable the recognition of diagnostic features often overlooked in traditional descriptions. Going forward, comprehensive and standardized documentation of dental crown variation across developmental stages and sexes will be vital for advancing our understanding of dental diversity and its evolutionary significance within the most diverse order of sharks.

## AUTHOR CONTRIBUTIONS

**Flávia Zanini:** Conceptualization; investigation; writing – original draft; methodology; funding acquisition; writing – review and editing. **Karla D. A. Soares:** Writing – review and editing; supervision; conceptualization; investigation; writing – original draft; funding acquisition.
